# Developmental predictors of suicide attempts from childhood to early adulthood: a 15-year prospective cohort study

**DOI:** 10.1016/j.lana.2026.101531

**Published:** 2026-06-25

**Authors:** Rodolfo Furlan Damiano, Daniel M. Low, Lucas Toshio Ito, Marcos Leite Santoro, Síntia Belangero, Pedro Mario Pan, Caio Casella, Julia Luiza Schäfer, Rudolf Uher, Peter Szatmari, Craig J. Bryan, Hilary P. Blumberg, Eurípedes Constantino Miguel, Luis Augusto Rohde, Giovanni Abrahão Salum

**Affiliations:** a Instituto de Psiquiatria, Hospital das Clínicas da Faculdade de Medicina da Universidade de São Paulo HCFMUSP, São Paulo, SP, Brazil; b Program for Education, Research, and Care in Treatment-Resistant Depression, Self-Injury, and Suicidality, IPq-HCFMUSP, São Paulo, SP, Brazil; c Center of Research and Innovation in Mental Health, CISM, Brazil; d Child Mind Institute, New York, NY, USA; e Department of Psychology, Harvard University, Cambridge, USA; f Molecular Biology Discipline, Universidade Federal de São Paulo, São Paulo, Brazil; g Graduate Program in Structural and Functional Biology, Universidade Federal de São Paulo, São Paulo, Brazil; h Department of Psychiatry, Universidade Federal de São Paulo, São Paulo, Brazil; i Department of Psychiatry, Dalhousie University, Halifax, NS, Canada; j Centre for Addiction and Mental Health, University of Toronto, Toronto, ON, Canada; k Department of Psychiatry, University of Vermont College of Medicine, Burlington, VT, USA; l Departments of Psychiatry, Radiology and Biomedical Imaging, and the Child Study Center, Yale School of Medicine, New Haven, CT, USA; m Departamento de Psiquiatria e Medicina Legal, Universidade Federal do Rio Grande do Sul, Porto Alegre, RS, Brazil

**Keywords:** Suicide attempt, Adolescence, Childhood adversity, Polygenic risk score, Intergenerational transmission, Low- and middle-income countries

## Abstract

**Background:**

Suicide attempts in childhood and adolescence are common, recurrent, and the strongest predictor of death by suicide; yet their developmental antecedents remain poorly understood, particularly in low- and middle-income countries. We aimed to identify childhood factors associated with incidence, onset timing, and number of suicide attempts and to quantify the population impact of modifiable risk factors.

**Methods:**

We analyzed data from the Brazilian High-Risk Cohort Study, a community-based cohort of 2511 children aged 6–14 years followed for 15 years. Risk factors across genetic, perinatal, sociodemographic, family, adversity, cognitive, and clinical domains were examined using logistic regression (OR), Cox proportional hazards (HR), and quasi-Poisson models (IRR), were examined for three outcomes (cumulative incidence, onset timing, and number of suicide attempts), with all predictors entered simultaneously and bootstrap resampling. Population attributable fractions were calculated for modifiable factors.

**Findings:**

Of 2060 participants (48.0% female unweighted, 47.3% weighted), 309 (15.0%) reported suicide attempts over 24,756.5 person-years (mean onset 17.8 years). Female sex (OR 3.06, 95% CI 2.28–4.17; HR 2.88, 2.25–3.69; IRR 3.12, 2.19–4.49) and childhood threat (OR 1.26, 1.08–1.47; HR 1.21, 1.06–1.38; IRR 1.26, 1.05–1.51) were associated with all three outcomes. Caregiver suicide attempt (OR 2.07, 1.40–3.14; HR 1.88, 1.34–2.63) and externalizing disorders (OR 1.52, 1.05–2.15; HR 1.50, 1.07–2.08) were associated with incidence and earlier onset. PRS-depression (IRR 1.39, 1.00–1.94) and gestational diabetes (IRR 2.18, 1.13–3.83) were associated with increased attempt frequency. Child-reported bullying (OR 1.73, 1.22–2.45) and parent-reported physical abuse (OR 1.96, 1.24–3.10) retained association after mutual adjustment in threat-component models. Population attributable fractions were substantial for caregiver suicide attempt (14.1%), caregiver internalizing disorders (11.6%), high childhood threat (>1 SD; 11.5%), and externalizing disorders (10.2%). In lethality analyses, PRS-depression (OR 2.21, 1.09–4.43; HR 1.98, 1.13–3.60; IRR 2.68, 1.43–4.97), childhood threat (HR 1.40, 1.09–1.80; IRR 1.34, 1.03–1.90), and maternal alcohol use in pregnancy (HR 1.92, 1.12–3.26) were associated with medically serious attempts. Prediction models showed modest discrimination (mean area under the curve [AUC] 0.665, SD 0.033, across 200 repeated test splits).

**Interpretation:**

These findings highlight three potentially modifiable or clinically actionable domains — childhood threat, caregiver suicidal behaviour history, and childhood externalizing disorders — that may be amenable to prevention even when genetic liability is present. Despite multi-domain predictors, predictive performance remained limited, consistent with a complementary role for population-level prevention alongside efforts to improve risk prediction.

**Funding:**

CNPq, FAPESP, ERC, UK MRC, and Banco Industrial do Brasil S/A. Full grant details are provided in the manuscript.

**Research in context**

**Evidence before this study**

We searched PubMed from database inception to Feb 19, 2026, for studies addressing developmental and familial predictors of suicide attempts in children, adolescents, and young adults. We used different combinations of the following terms: “suicide attempt”, “attempted suicide”, “self-harm”, “suicidal behaviour”, “suicidal behavior”, “suicidality”, “longitudinal cohort”, “prospective cohort”, “birth cohort”, “childhood”, “adolescence”, “adolescent”, “young adult”, “risk factor”, “predictor”, “developmental”, “polygenic risk score”, “GWAS”, “family history”, “intergenerational”, “perinatal”, “childhood adversity”, “childhood maltreatment”, “bullying”, “externalizing”, “internalizing”, “Latin America”, “Brazil”, and “low- and middle income countries”. No language restrictions were applied. We additionally hand-searched the reference lists of relevant systematic reviews, meta-analyses, and included primary studies. Previous longitudinal and meta-analytic evidence has identified several childhood and familial factors associated with later suicide attempts, including female sex, childhood maltreatment, parental psychopathology, family history of suicidal behaviour, and early-onset psychiatric disorders. More recent work has also examined polygenic liability, but genetic studies of suicidal behaviour and related psychiatric phenotypes have been conducted predominantly in adult and European-ancestry populations, with limited portability to admixed or underrepresented populations. Overall, the available evidence remains concentrated in high income settings and is often organised around single risk domains or single outcome definitions. Prospective evidence from Latin America is scarce, and few studies have evaluated how genetic, familial, perinatal, adversity-related, cognitive, and clinical factors compare when assessed in childhood and followed into early adulthood.

**Added value of this study**

Prospective cohort studies of suicide attempts with long-term follow-up remain rare globally and are particularly scarce in Latin America, where suicide rates among young people have been rising. To our knowledge, this study is among the first from the region to jointly examine polygenic, familial, perinatal, and childhood-adversity factors as candidate predictors of adolescent suicide attempts across three complementary outcomes — cumulative incidence, onset timing, and number of attempts — within a single prospective cohort followed for approximately 15 years. By integrating biological, familial, and environmental domains in a community-based sample of 2060 Brazilian children, the study contributes evidence on: (i) which factors are robustly associated with multiple outcomes vs those that appear outcome-specific; (ii) the persistence of an intergenerational association between caregiver suicidal behaviour and offspring suicide attempts after adjustment for polygenic liability and general parental psychopathology; and (iii) the differential pattern of factors associated with medically serious attempts, including childhood threat and perinatal exposures. These findings extend the evidence base for developmental predictors of suicide attempts beyond high-income settings.

**Implications of all the available evidence**

Together with prior evidence, the findings are consistent with multi-level suicide prevention strategies that consider population-level impact alongside high-risk approaches. The association between caregiver suicidal behaviour and offspring risk is consistent with addressing parental mental health and family history as part of prevention efforts, with potential downstream benefits for offspring. Childhood externalizing disorders are associated with risk and may warrant attention when assessing suicide risk in youth presenting with behavioural problems, in addition to internalizing symptoms. At the population level, childhood threat — and particularly bullying — was associated with suicide attempt incidence, consistent with the relevance of school-based prevention. The observation that perinatal factors — including alcohol use in pregnancy and markers of high-risk pregnancy — were associated with medically serious attempts is consistent with a developmental view of severe suicidal behaviour and suggests that maternal healthcare may be relevant as an upstream component of prevention. Although biological factors, including PRS-depression, were associated with some outcomes, their predictive utility on an individual level remains modest, suggesting that reductions in suicide attempts are more likely to be achieved through broad prevention strategies than through risk stratification alone. Continued work on prediction models may help refine high-risk approaches and inform more precise interventions when risk is imminent.

## Introduction

Suicide is a leading cause of death among adolescents and young adults worldwide and remains a major public health challenge.^1^ Although a substantial proportion of suicides occur during a person's first attempt,^2^ underscoring the challenge of identifying high-risk individuals before suicidal behaviour ever manifests, prior suicide attempts are the most robust individual risk factor.^3^ This finding places suicide attempts at the centre of prevention efforts at two levels: the population and the individual. At the population level, it is important to understand which modifiable risk factors should be the focus of broad policies. At the individual level, understanding who attempts suicide and why is essential for identifying individuals at the highest risk before a fatal outcome occurs.

Research over the past two decades has identified several domains of risk factors that precede suicidal behaviour. At the biological level, family and twin studies indicate moderate heritability of suicide attempts (30–50%), and genome-wide association studies have begun to identify specific genetic variants that contribute to this liability.^4^ At the environmental level, childhood adversity, particularly experiences of abuse, neglect, and family dysfunction, consistently emerges among the strongest predictors, with meta-analyses demonstrating two-to three-fold increases in risk following maltreatment.^5^ At the individual level, psychiatric disorders represent well-established risk factors, including both internalizing conditions such as depression and anxiety, and externalizing conditions such as attention-deficit/hyperactivity disorder and conduct problems.^6^ Cognitive factors, including deficits in executive function and problem-solving, may contribute to risk by impairing the ability to generate alternatives to suicide during crises.^7^^,^^8^ At the perinatal level, a systematic review and meta-analysis found that adverse in-utero conditions are associated with increased suicide risk across the lifespan, supporting a developmental origins framework for suicidal behaviour.^9^ Finally, at the family level, parental psychopathology and family history of suicidal behaviour contribute to intergenerational transmission of risk through both genetic and environmental pathways.^10^ Taken together, this evidence suggests that suicide attempts arise from the confluence of genetic vulnerability, adverse early environments, psychopathology, and family context.

Despite this substantial body of research, critical gaps limit our ability to translate these findings into effective prevention. First, most studies have examined risk factors in isolation, leaving uncertainty about which predictors retain independent effects when considered simultaneously. Second, prior longitudinal research has been limited, and the vast majority has come from high-income countries, raising questions about generalisability to low- and middle-income countries (LMICs), where social and environmental contexts differ markedly and where the burden of youth suicide is concentrated.^11^, ^12^, ^13^ Third, few studies have integrated genetic risk scores with environmental and clinical predictors within a developmental framework, limiting our understanding of how these factors combine to shape risk trajectories from childhood through adolescence. Fourth, while theoretical models such as the Dimensional Model of Adversity propose that threat-related experiences (e.g., abuse, violence) and deprivation-related experiences (e.g., neglect, poverty) operate through distinct mechanisms,^14^ empirical tests of whether these dimensions differentially predict suicidal behaviour remain scarce.

The present study addresses these gaps using data from the Brazilian High-Risk Cohort for Mental Health Conditions (BHRC), a 15-year prospective cohort of children aged 6–14 years with 82% retention.^15^ Our multi-domain variable selection is informed by contemporary ideation-to-action theories—the Interpersonal-Psychological Theory, the Integrated Motivational-Volitional model, and the Three-Step Theory^16^, ^17^, ^18^, ^19^—which distinguish motivational processes driving suicidal ideation from volitional processes enabling the transition to action. In this framework, adversity and family environments may foster psychological pain and thwarted belongingness (motivational), while genetic and perinatal factors may index dispositional capability (volitional). We examined factors associated with suicide attempts across seven domains: genetic, sociodemographic, perinatal, childhood adversity, cognitive, clinical, and family. Employing complementary analytical approaches, our objective was to identify childhood factors associated with suicide attempts after mutual adjustment in multivariable models and to estimate the population-level impact of specific threat exposures.

## Methods

### Sample

The BHRC is a community-based school cohort recruited from 57 state-funded schools in Porto Alegre and São Paulo, Brazil. At screening (2009–2010), 9937 children aged 6–14 years were assessed using the Family History Screen. From this sample, 2511 participants were selected for deep phenotyping: 958 randomly selected and 1553 based on a high-risk procedure identifying children with psychiatric symptoms and/or elevated family history of mental disorders. Although schools were drawn from the public education system, which predominantly serves lower-income communities, the inclusion of multiple schools across two Brazilian cities ensured representation of diverse socioeconomic areas. The high-risk score was calculated as a family liability index expressing the percentage of first-degree relatives who screened positive for psychiatric disorders. Baseline assessment (W0) occurred in 2010–2011 (mean age 10.4 years, SD 1.9). Participants were reassessed at Wave 1 (2013–2014; mean age 13.8 years), Wave 2 (2018–2019; mean age 18.3 years), and Wave 3 (2023–2025; mean age 23.2 years), spanning middle childhood through early adulthood.

All procedures adhered to the Helsinki Declaration (revised 2008) and were approved by institutional review boards (CAAE: 74563817.7.1001.5237 and 74563817.7.2002.5505). Written informed consent was obtained from all adult participants. For those under 18 years old, informed consent was obtained from a parent or legal guardian, together with child assent. For detailed procedures, see Salum et al. 2025.^15^ This study is reported in accordance with the Strengthening the Reporting of Observational Studies in Epidemiology (STROBE) guidelines for cohort studies.^20^ The completed STROBE checklist is provided as a separate file accompanying this submission.

### Measures

#### Outcomes

##### Columbia-Suicide Severity Rating Scale (C-SSRS)

Suicide attempts were assessed in Wave 3 using the Columbia-Suicide Severity Rating Scale (C-SSRS), a semi-structured interview designed to categorise the severity of suicidal ideation and behaviour. There were no recorded suicide deaths in the cohort prior to attempt assessment. The scale distinguishes between suicidal ideation (passive death wish, active ideation with or without intent, ideation with plan) and suicidal behaviour (preparatory acts, aborted attempts, interrupted attempts, and actual attempts), with demonstrated sensitivity and specificity for identifying suicidal behaviours in clinical and research settings.^21^ We used the following items to assess three different outcomes: (a) suicide attempt: “*Did you do anything to try to kill yourself or make yourself not alive anymore?*”; (b) total number of actual attempts (lifetime); and (c) date of the first attempt combined with interview age. The C-SSRS distinguishes between suicidal behaviour and non-suicidal self-injurious behaviour based on the presence of intent to die. Suicide attempts were defined as self-injurious behaviour performed with at least some intent to die, as determined by the C-SSRS interviewer. Deliberate self-harm without suicidal intent (i.e., non-suicidal self-injury) was identified by the absence of any reported intent to die and was excluded from the outcome definition. This distinction was made by trained interviewers following the C-SSRS administration protocol (https://cssrstraining.com/). For all incident-attempt analyses, participants whose first suicide attempt occurred before baseline were excluded; non-suicidal self-injury at baseline was retained as a separate covariate (recent DSH/suicidal talk) and not used as a longitudinal exclusion criterion. The C-SSRS has been validated in multiple populations, including adolescents as young as five years of age, and is recommended by the US Food and Drug Administration for clinical trials assessing suicide risk.^22^ Lifetime suicidal ideation, assessed via the C-SSRS at Wave 3, was not included as a covariate in the main prospective models because it was ascertained retrospectively and concurrently with the outcome, precluding temporal ordering; its inclusion would risk overadjustment for a proximal construct measured at the same assessment point. An ideation-restricted sensitivity analysis is presented separately.

### Risk factors

Sociodemographic characteristics included sex assigned at birth (male/female) and self-reported race/ethnicity, dichotomised as white vs non-white (including Black, mixed-race [pardo], Asian, and Indigenous), given the Brazilian context and sample distribution. Gender identity was not assessed in the first wave; all references to sex in this manuscript refer to sex assigned at birth. The following sections describe additional instruments and assessments employed in the present analyses. All risk factors were assessed only at baseline.

#### Development and Well-Being Assessment (DAWBA)

Psychiatric diagnoses were obtained using the Development and Well-Being Assessment (DAWBA),^23^^,^^24^ a comprehensive tool comprising questionnaires, interviews, and rating methods that provide DSM diagnoses for youth aged 5 to 17 and has been adapted for adult populations. In the BHRC, DAWBA clinical ratings showed high inter-rater agreement (kappa = 0.80 for any disorder, 0.72 for hyperkinetic disorders, 0.85 for emotional disorders, and 0.73 for conduct disorders; all p < 0.001). It uses a two-stage process: lay interviewers first ask structured questions, then follow up with open-ended prompts transcribed verbatim; trained clinicians then review all data to assign diagnoses. Participants completed caregiver interviews, and those 11+ also provided self-reports. We created two binary indicators of child psychopathology at baseline. The internalizing disorders variable included diagnoses such as major depression, generalized anxiety disorder, separation anxiety disorder, panic disorder, agoraphobia, social phobia, specific phobia, obsessive-compulsive disorder (OCD), post-traumatic stress disorder (PTSD), tics, eating disorders, or mania. The externalizing disorders variable covered attention-deficit hyperactivity disorder (ADHD), oppositional defiant disorder, or conduct disorder. Recent deliberate self-harm/suicidal talk at baseline (W0) was analysed using the binary question (yes/no) asking the parents whether the child “*has talked recently about deliberate self-harm (DSH)*”. Additionally, lifetime DSH was assessed across all four study waves using a binary item (yes/no) from the DAWBA.

#### Adversity experiences

Drawing on theories that distinguish threat from deprivation,^25^ we used BHRC baseline data to operationalise these constructs.^26^ The two-factor model demonstrated adequate fit in this sample (CFI = 0.937, TLI = 0.922, RMSEA = 0.032).^26^ Factor scores were used as continuous predictors. Threatening experiences were assessed using items from the DAWBA PTSD module and BHRC-specific questionnaires, which were administered to all participants regardless of PTSD diagnostic status.^15^^,^^23^ Lifetime data on physical and sexual abuse, attacks or threats, witnessing domestic violence, and witnessing attacks were derived from parental reports via DAWBA items, e.g., “*Has the child ever suffered physical violence (maltreatment) that he/she remembers?*” Other variables, such as lifetime bullying and abuse frequency, were collected from caregivers and children using questions such as “*Has the child (you) ever been bullied?*” and “*Has your child (you) ever been cursed or yelled at by an adult?*”.^27^

Deprivation included neglect, parental absence, and material hardship, linked to cognitive deprivation, such as limited early exposure to complex language.^14^^,^^25^^,^^28^ Indicators included maternal education (four levels), household income (quintiles), socioeconomic status per the Brazilian Economic Classification (categories A/B to D/E),^29^ paternal contact (in contact, no contact, deceased, unknown), and physical neglect (never, once or twice, occasionally, often).^27^ Physical neglect was assessed via parent and child reports using the item “*Has it ever happened to your child (you) of not having anything to eat and/or having to wear dirty or torn clothes?*” Paternal contact status was determined through the question “*What is the current contact status of the child's father?*”

#### Intelligence quotient

Intelligence quotient (IQ) was assessed using the vocabulary and block design subtests of the Wechsler Intelligence Scale for Children (WISC-III),^30^ a standardised measure designed for rapid assessment of cognitive ability using the Tellegen and Briggs (1967)^31^ method and Brazilian norms.^32^

#### Executive function assessment

Three executive function (EF) dimensions were calculated to develop a second-order EF model, following the model described in Martel et al. (2017).^33^ The dependent variable was a standardised EF score from working memory, inhibitory control, and temporal processing scores, after regressing out age and gender effects using General Additive Models. Higher scores indicate better EF. A second-order model was used, where EF was a higher-order factor encompassing three lower-order factors: working memory (Digit Span Backwards, Corsi Blocks Backwards), inhibitory control (Go/No-Go, Conflict Control), and temporal processing (Time Anticipation at 400 ms). Psychologists administered these tasks, and scores were converted to z-scores based on age norms before creating a composite EF measure.

#### Main caregiver psychiatric assessment

Caregiver psychopathology was assessed with the M.I.N.I. (Mini-International Neuropsychiatric Interview), a brief structured interview for psychiatric disorders based on DSM-IV and ICD-10 criteria.^34^ The M.I.N.I. has good reliability and validity and has been validated for Brazilian populations.^35^ Following the Hierarchical Taxonomy of Psychopathology (HiTOP) framework,^36^ we created three binary indicators: (1) internalising disorders (current panic disorder, agoraphobia, social anxiety disorder, generalised anxiety disorder, major depressive episode, or recurrent depression); (2) externalising disorders (current alcohol dependence or abuse, drug dependence or abuse, or ADHD); and (3) thought disorder (lifetime mania or psychotic disorder). Current diagnoses were used for internalising and externalising domains; lifetime diagnoses were used for thought disorder, as lifetime measures were not available for the remaining M.I.N.I. diagnoses in this dataset. The caregiver's lifetime suicide attempt, assessed at baseline, was evaluated via the M.I.N.I. suicidality module with the question “Have you ever attempted to kill yourself?” and coded as yes/no.

#### Polygenic risk scores

Polygenic risk scores (PRS) were calculated using summary statistics from genome-wide association studies (GWAS) of relevant psychiatric phenotypes. Genotype data were generated using the Illumina Global Screening Array and underwent standard quality control, excluding variants with minor allele frequency <1%, call rate <98%, Hardy–Weinberg equilibrium [HWE] p < 1 × 10^−10^, as well as samples with call rate <98%. Imputation was performed using the TOPMed reference panel,^37^ retaining only high-quality variants (imputation R^2^ > 0.8). Genetic principal components were calculated using PC-AiR,^38^ and the first ten principal components were included as covariates in all models to adjust for population stratification. PRS were computed using PRS–CS–auto^39^ with default settings, using external GWAS summary statistics to derive single-nucleotide polymorphism (SNP) weights independently of the present sample. PRS were included for the following phenotypes using the latest GWAS summary statistics: (1) major depressive disorder^40^; (2) anxiety disorders^41^; (3) subjective well-being^42^; (4) suicide attempt^43^; and (5) suicide death.^44^ All scores were computed in PLINK^45^ and standardised (mean = 0, SD = 1) prior to analysis.

#### Prenatal and perinatal risk factors

Prenatal and perinatal information was obtained through structured questionnaires administered to the participant's primary caregiver during household interviews conducted by trained interviewers. When available, gestational and birth information was confirmed using the child's health chart records. All variables were coded as binary indicators to minimise recall bias and maximise clinical interpretability.^15^^,^^46^

Maternal substance use during pregnancy included tobacco exposure, dichotomised as moderate-to-heavy (≥1 cigarette/day) vs none/minimal, and any alcohol consumption vs none. Birth and neonatal outcomes comprised low birthweight (<2500 g), prematurity (<37 weeks of gestation), and neonatal intensive care unit admission. Pregnancy complications included eclampsia, gestational diabetes, urinary tract infection, and other significant complications. Prenatal and postnatal care indicators included adequate prenatal care (≥8 visits, following Brazilian Ministry of Health guidelines) and any breastfeeding vs none.

### Statistical analysis

We employed three complementary analytical approaches to capture distinct aspects of suicide attempt risk: logistic regression for cumulative incidence, Cox proportional hazards for modelling the hazard of first attempt as a function of age, and quasi-Poisson regression for number of attempts. Single-predictor analyses examined each risk factor separately with false discovery rate (FDR) correction applied across risk factors within each model. Multivariable analyses included all risk factors simultaneously, with bootstrap resampling (1000 iterations) on the log-coefficient scale; point estimates are means of the bootstrap distribution (exponentiated), with 95% confidence interval (CI) from the 2.5th–97.5th percentiles of log-scale estimates (exponentiated). Convergence failures, Monte Carlo standard error of CI endpoints, and bootstrap distribution skewness are reported in Table S1. To quantify the incremental contribution of each risk factor domain, we fit sequential weighted logistic models adding domains cumulatively in temporal order (sociodemographic → adversity → family → clinical → perinatal → cognitive → genetic), reporting Nagelkerke pseudo-R^2^ at each step. A directed acyclic graph (DAG; Figure S1) presents the hypothesised causal structure across temporal layers, clarifying which variables function as confounders vs potential mediators. Because all predictors are entered simultaneously, multivariable coefficients represent associations after mutual adjustment (analogous to direct effects in a causal framework, though observational associations do not establish causality); single-predictor estimates capture total associations, including mediated pathways. Prior to multivariable modelling, we assessed multicollinearity using generalised variance inflation factors (GVIF; computed from the logistic regression model using car::vif in R version 4.5.2, which yields GVIFˆ(1/2Df) for categorical predictors; Table S2) and a Spearman correlation heatmap (Figure S2). All GVIF values were below 5 (only PRSs were above 2), indicating acceptable collinearity levels. Spearman correlations were generally low (median r = 0.05; 93.7% with |r| < 0.3). Higher correlations (|r| > 0.5) were observed exclusively among polygenic risk scores (range: 0.61–0.75).

**Logistic regression** for any incident attempt (binary outcome) using survey-weighted generalised linear models with quasibinomial family, yielding odds ratios (OR).

**Cox proportional hazards regression** for time to first suicide attempt, using age as the time scale with left truncation (delayed entry) to account for varying ages at study enrolment, yielding hazard ratios (HR). Sampling weights were incorporated via robust variance estimation with clustering by participant ID. A total of 325 participants reported at least one lifetime suicide attempt on the C-SSRS; 16 whose first attempt preceded baseline were excluded from incident-attempt analyses, yielding 309 incident attempters for logistic and quasi-Poisson analyses. For Cox models, timing of first attempt was required: 245 of the 325 attempters provided specific dates on the C-SSRS, 69 provided timing imputed from the earliest wave with endorsed deliberate self-harm on the DAWBA, and 11 had no time information from either source and were excluded from Cox analyses. The Cox analytic sample therefore comprised 2049 participants with 298 events. As a sensitivity analysis, Cox models were restricted to attempters with confirmed C-SSRS dates only. Of the 245 lifetime attempters with C-SSRS dates, 229 remained as incident attempters in this restricted Cox sensitivity sample (Cox sample N = 1980; Table S3) after excluding pre-baseline attempts. The proportional hazards assumption was tested using Schoenfeld residuals. The global test indicated non-proportionality; reported hazard ratios are interpreted as weighted average effects across the follow-up period (see Limitations).

**Quasi-Poisson regression** for the number of incident attempts using survey-weighted generalised linear models, yielding incidence rate ratios (IRR). The quasi-Poisson family was preferred over negative binomial regression because it accommodates overdispersion without imposing a specific mean–variance relationship and is compatible with survey weighting via the quasipoisson family in R. The estimated dispersion parameter was ϕ = 10.4, confirming substantial overdispersion.

To address oversampling of high-risk participants and differential attrition, we applied covariate-balancing propensity score (CBPS) weights to recover the covariate distribution of the full screening sample. The CBPS model included state, child age, child sex, number of biological and half-siblings, and family liability indices for ADHD, anxiety, OCD, psychosis, and learning disorders from both the screened child and non-screened siblings. Weight distribution, effective sample size, standardised mean differences before and after weighting, and sensitivity to weight trimming are reported in Table S4. These weights target the covariate distribution of the full screening sample (N = 9937), enabling population-representative inference for the school-attending population in these cities. Multiple imputation using chained equations (MICE) addressed missing risk factor data (m = 5 imputations, 10 iterations), using predictive mean matching for continuous and logistic regression for binary variables; auxiliary variables included age, selection status, CBPS weights, and outcome indicators. Per-variable missingness is reported in Table S5. Results were pooled using Rubin's rules. To address potential bias from differential attrition, we estimated inverse probability of attrition weights (IPAW) using Wave 3 completion status in the original deeply phenotyped cohort (451 non-completers among N = 2511), and then applied combined CBPS × IPAW weights to the incident-risk analytic sample (N = 2060). Main models were re-run with these combined weights as a sensitivity analysis (Table S6). Polygenic risk score analyses included 10 genetic principal components as covariates. As a sensitivity analysis, we computed the first principal component (PC1) of the five PRS as a composite genetic liability score capturing 63.6% of total PRS variance and re-fit all three models substituting PC1 for the individual PRS (Table S7). The collective contribution of the PRS block is reported in the sequential model-building analysis (Table S8). PC1 captured 63.6% of total PRS variance (loadings: depression 0.52, anxiety 0.50, suicide attempt 0.51, suicide death 0.38, well-being −0.27). To quantify population-level impact of modifiable risk factors, we calculated population attributable fractions (PAF) using Levin's formula, PAF = P_pop (OR−1)/[1 + P_pop (OR−1)], where P_pop is the prevalence of exposure in the analytic sample, with odds ratios adjusted for sex and age only, because minimally adjusted estimates better approximate the total population impact, including both direct and mediated effects; PAFs using fully adjusted ORs are presented as a sensitivity analysis (Table S9). Bootstrap resampling (1000 iterations) provided 95% confidence intervals. For childhood threat, sensitivity to alternative thresholds (>0.5 SD, >1.0 SD, >1.5 SD) is reported in Table S9. Because the cumulative incidence of suicide attempts in this cohort (15.0%) is not rare, OR-based Levin PAFs may slightly overestimate risk-ratio-based attributable fractions; PAFs are therefore interpreted as approximate population-impact metrics under assumptions of causality, no residual confounding, and no interaction among exposures, rather than directly preventable proportions of cases.

Several supplementary analyses extended and validated our main findings. These included: sample characteristics comparing the full cohort to the analytic sample; unweighted models to verify robustness to CBPS weighting; benchmarking logistic regression against machine learning classifiers (Elastic Net, Random Forest, XGBoost, Support Vector Machine) with precision–recall metrics for imbalanced data; threat component analyses stratified by informant source; population attributable fractions for specific threat components; high-lethality analyses comparing high-lethality attempters to non-attempters; a within-attempter analysis comparing high-lethality (physical damage ≥2) vs low-lethality (score 0–1) attempters; and an ideation-restricted sensitivity analysis comparing attempters vs ideators-without-attempt, excluding participants with no lifetime suicidal ideation, to test whether risk factor associations are specific to the transition from ideation to action or shared with ideation risk. For machine learning analyses, we performed 200 repeated stratified 80/20 splits,^47^^,^^48^ reporting mean ± SD of ROC AUC and PR AUC across splits for each model (logistic regression, Elastic Net, Random Forest, XGBoost, SVM). Class weighting (inverse class frequency) was applied during training; the natural class distribution was preserved in each test set. Hyperparameters were tuned via 5-fold CV on the initial training set and held fixed across splits (Table S10). These analyses were intended as pragmatic benchmarking rather than formal clinical prediction model development. Detailed methods for all supplementary analyses are provided in the Supplementary Methods.

All analyses were conducted in R version 4.5.2 (R Foundation for Statistical Computing) using key packages including survey, survival, mice, CBPS, glmnet, and randomForest. Statistical significance was set at two-sided p < 0.05 for single-predictor tests and FDR-corrected q < 0.05 for multiple comparisons; for multivariable bootstrap analyses, significance was determined when 95% confidence intervals excluded 1.00.

### Role of funding source statement

The funders of the study had no role in study design, data collection, data analysis, data interpretation, or writing of the report. All authors agreed on the final version of the manuscript, and the decision to submit the manuscript for publication was made independently by the first and last authors (RFD and GAS), who also selected the journal.

## Results

### Sample characteristics

Of the 2511 participants assessed at the baseline wave (Wave 0) of the deeply phenotyped cohort, 2495 had complete baseline data after excluding pre-baseline attempts. Of these, 2060 (82.6%) completed the Wave 3 assessment and comprised the analytic sample for logistic and quasi-Poisson models; 435 (17.4%) were lost to follow-up over the 15-year follow-up period. When considered with reference to the full deeply phenotyped cohort, 451/2511 (18.0%) participants did not complete Wave 3; this denominator was used for inverse probability of attrition weighting (see Methods). For Cox regression, an additional 11 participants were excluded due to missing survival time variables, yielding a final sample of 2049 (Table S11). The sample was balanced by sex, with 989/2060 participants female (48.0% unweighted; 47.3% weighted using CBPS), with a mean baseline age of 10.4 years (SD 1.9) and a mean age at Wave 3 of 23.2 years (SD 1.9). Baseline psychopathology was common (Table 1): 12.4% met criteria for internalising disorders, 15.2% for externalising disorders, and 3.1% for both. Among caregivers, 30.5% met criteria for at least one internalising disorder, 1.1% for externalising, and 0.9% for both. Additionally, 11.9% of caregivers met criteria for thought disorder, and the proportion of caregivers with co-occurring conditions is reported in Table 1. Self-reported race/ethnicity included white (54.4%), mixed-race/Pardo (31.1%), Black/African-Brazilian (14.2%), and Asian/Indigenous (0.4%). Unless otherwise stated, descriptive percentages in the text are weighted using CBPS weights to recover the covariate distribution of the full screening sample (N = 9937); corresponding unweighted n and percentages are reported in Table 1. Cohort-flow statistics, the analytic- vs -enrolled cohort comparison below, and within-attempter distributions are reported as observed (unweighted) sample proportions.

**Table 1:** Baseline characteristics of the study sample by suicide attempt status.

Characteristic	Unweighted			Weighted (%)			p-value
	Overall	No attempt	Attempt	Overall	No Attempt	Attempt	
Genetic							
PRS Depression (z)	0.01 (0.99)	−0.02 (0.99)	0.14 (1.02)	0.03 (1.00)	−0.02 (0.97)	0.30 (1.08)	0.002
PRS Anxiety (z)	0.01 (1.00)	−0.01 (1.00)	0.12 (0.99)	0.02 (1.00)	−0.02 (0.99)	0.26 (1.02)	0.002
PRS Well-being (z)	−0.00 (1.01)	0.02 (1.00)	−0.14 (1.04)	−0.06 (1.04)	−0.02 (1.03)	−0.26 (1.09)	0.013
PRS Suicide Attempt (z)	0.00 (0.99)	−0.01 (0.99)	0.08 (0.96)	0.01 (0.99)	−0.01 (0.99)	0.15 (0.95)	0.031
PRS Suicide Death (z)	0.02 (1.01)	0.02 (1.01)	−0.01 (0.98)	0.05 (1.01)	0.04 (1.02)	0.10 (1.00)	0.510
Perinatal							
Maternal smoking in pregnancy	334 (16.2)	270 (15.4)	64 (20.7)	16.4	15.1	23.8	0.006
Maternal alcohol use in pregnancy	451 (21.9)	362 (20.7)	89 (28.8)	21.4	20.1	28.6	0.009
Low birthweight (<2500 g)	219 (10.6)	184 (10.5)	35 (11.3)	10.7	10.2	13.4	0.237
Prematurity	309 (15.0)	262 (15.0)	47 (15.2)	15.1	15.3	13.7	0.552
Breastfeeding	1890 (91.7)	1602 (91.5)	288 (93.2)	91.6	91.5	92.2	0.748
Prenatal care ≥8 visits	1454 (70.7)	1241 (70.9)	213 (69.4)	70.0	69.4	73.3	0.263
Neonatal ICU admission	234 (11.4)	210 (12.0)	24 (7.8)	11.3	11.4	10.6	0.798
Eclampsia	404 (19.6)	344 (19.6)	60 (19.4)	19.9	20.2	17.8	0.418
Gestational diabetes	90 (4.4)	69 (3.9)	21 (6.8)	4.2	3.7	7.2	0.030
UTI in pregnancy	396 (19.2)	334 (19.1)	62 (20.1)	22.0	21.6	24.7	0.415
Other pregnancy complication	113 (5.5)	97 (5.5)	16 (5.2)	6.2	6.0	7.8	0.497
Sociodemographic							
Female sex	989 (48.0)	776 (44.3)	213 (68.9)	47.3	43.2	70.3	<0.001
Non-white race	904 (43.9)	767 (43.8)	137 (44.3)	45.6	45.2	48.0	0.483
Family (Main Caregiver)							
Caregiver internalising disorder	598 (29.0)	486 (27.8)	112 (36.2)	30.5	28.9	39.8	0.005
Caregiver externalising disorder	26 (1.3)	24 (1.4)	2 (0.6)	1.1	1.2	0.6	0.373
Caregiver suicide attempt	251 (12.2)	186 (10.6)	65 (21.0)	13.1	11.5	22.6	<0.001
Caregiver thought disorder	216 (10.5)	170 (9.7)	46 (14.9)	11.9	11.0	17.1	0.049
Adversity							
Threat exposure (z)	−0.00 (1.00)	−0.05 (0.98)	0.27 (1.10)	0.01 (1.03)	−0.05 (0.98)	0.38 (1.20)	<0.001
Deprivation (z)	−0.03 (1.00)	−0.05 (0.99)	0.09 (1.03)	−0.04 (0.99)	−0.06 (0.99)	0.08 (1.00)	0.055
Cognitive							
IQ (z)	0.03 (1.00)	0.03 (1.00)	0.02 (0.95)	0.02 (0.98)	0.03 (0.99)	−0.00 (0.94)	0.674
Executive function (z)	0.01 (0.99)	0.01 (0.99)	0.03 (0.95)	0.02 (0.99)	0.01 (1.00)	0.05 (0.96)	0.637
Clinical (W0)							
Any internalising disorder	261 (12.7)	211 (12.1)	50 (16.2)	12.4	11.8	15.5	0.172
Any externalising disorder	285 (13.8)	226 (12.9)	59 (19.1)	15.2	14.1	21.2	0.037

N: Overall = 2060; No Attempt = 1751; Attempt = 309. Unweighted: n (%) for categorical, mean (SD) for continuous variables. Weighted: % for categorical, mean (SD) for continuous variables (using CBPS weights). PRS = polygenic risk score; z = standardized score. p-values from weighted chi-square test (categorical) or weighted regression (continuous).

The analytic sample (N = 2060) did not differ from the total enrolled cohort (N = 2511) regarding sex distribution (989/2060 [48.0%] vs 1135/2511 [45.2%] female, p = 0.06), race (904/2060 [43.9%] vs 1103/2511 [43.9%] non-white, p = 0.99), childhood adversity (threat and deprivation, both p > 0.35), cognitive measures (IQ and executive function, both p > 0.50), polygenic risk scores (all p > 0.59), baseline psychopathology (internalising disorder 261/2060 [12.7%] vs 302/2511 [12.0%], p = 0.54; externalising disorder 285/2060 [13.8%] vs 353/2511 [14.1%], p = 0.86), or caregiver characteristics (all p > 0.50). These findings suggest that the final analytic sample remained broadly comparable to the enrolled cohort on measured baseline characteristics (Table S12).

### Prevalence and distribution of suicide attempts

Over 24,756.5 person-years of observation (mean follow-up 12.1 years per participant), 309 participants (15.0%) reported at least one incident suicide attempt. Among those with timing information (n = 298), the incidence rate was 12.04 per 1000 person-years (95% CI 10.7–13.4). Incidence peaked during mid-adolescence (ages 14–17: 16.2–16.5 per 1000 person-years) before declining in early adulthood (ages 18–21: 11.6–11.8 per 1000 person-years). The mean age at first attempt was 17.8 years (SD 3.7; range 10.1–26.9). Female participants presented a markedly higher prevalence than males (22.3% vs 8.5%; p < 0.001).

A total of 774 attempts were reported among the 309 individuals who attempted, yielding a mean of 2.5 attempts (SD 3.2; median 1; range 1–25). The majority reported a single attempt (175, 56.6%), 55 (17.8%) reported two, and 79 (25.6%) reported three or more. A subset of 21 individuals (6.8%) reported ≥10 attempts, accounting for 33% of all attempts, highlighting the concentration of repeat attempts among high-risk individuals. Physical damage was assessed using the C-SSRS lethality subscale (scores 0–5). Of those with available data (n = 287, 92.9%), 116 (40.4%) sustained no physical damage, 80 (27.9%) had minor physical damage, and 91 (31.7%) were classified as high-lethality attempters, including 57 with moderate physical damage (score 2), 21 with moderately severe damage, and 13 with severe damage.

### Risk factors for suicide attempts

Table 2 presents single-predictor and multivariable results from bootstrap models including all candidate risk factors simultaneously. Female sex was the strongest and most consistent risk factor across all three outcomes: occurrence (OR 3.06, 95% CI 2.28–4.17), onset timing (HR 2.88, 95% CI 2.25–3.69), and number of attempts (IRR 3.12, 95% CI 2.19–4.49). Childhood threat remained associated with all outcomes after mutual adjustment: occurrence (OR 1.26, 95% CI 1.08–1.47), earlier onset (HR 1.21, 95% CI 1.06–1.38), and number of attempts (IRR 1.26, 95% CI 1.05–1.51). Caregiver suicide attempt was associated with occurrence (OR 2.07, 95% CI 1.40–3.14) and earlier onset (HR 1.88, 95% CI 1.34–2.63), but not with the number of attempts. Baseline externalising disorders retained association with incident attempts in multivariable models (OR 1.52, 95% CI 1.05–2.15) and earlier onset (HR 1.50, 95% CI 1.07–2.08). Among perinatal factors, maternal alcohol use in pregnancy was associated with earlier onset (HR 1.35, 95% CI 1.01–1.77), while prematurity showed an unexpected protective association with the number of attempts (IRR 0.57, 95% CI 0.32–0.93). Gestational diabetes was associated with the number of attempts (IRR 2.18, 95% CI 1.13–3.83). Polygenic risk scores showed outcome-specific associations: higher PRS-depression was associated with the number of attempts (IRR 1.39, 95% CI 1.00–1.94), while PRS-suicide death showed an inverse association with the number of attempts (IRR 0.73, 95% CI 0.57–0.92).

**Table 2:** Single-predictor and multivariable bootstrap associations between childhood risk factors and suicide attempts (cumulative incidence, onset timing, and attempt frequency).

Variable	Single-predictor			Multivariable (Bootstrap)		
	LR (OR)	Cox (HR)	QP (IRR)	LR (OR)	Cox (HR)	QP (IRR)
Genetic						
PRS Depression (z)	1.26 (1.08–1.46)∗∗^,^†	1.23 (1.06–1.41)∗∗^,^†	1.18 (0.96–1.44)	1.22 (0.89–1.65)	1.24 (0.96–1.61)	1.39 (1.00–1.94)
PRS Anxiety (z)	1.18 (1.03–1.37)∗	1.14 (1.00–1.30)	1.05 (0.87–1.27)	1.03 (0.78–1.32)	0.97 (0.77–1.23)	0.77 (0.56–1.06)
PRS Wellbeing (z)	0.84 (0.74–0.95)∗	0.87 (0.78–0.97)∗^,^†	0.97 (0.82–1.15)	0.88 (0.76–1.03)	0.91 (0.79–1.04)	1.03 (0.88–1.23)
PRS Suicide Attempt (z)	1.13 (0.96–1.33)	1.10 (0.94–1.29)	1.10 (0.90–1.38)	0.88 (0.64–1.18)	0.88 (0.65–1.21)	1.18 (0.79–1.75)
PRS Suicide Death (z)	1.01 (0.86–1.19)	1.00 (0.87–1.16)	0.88 (0.71–1.10)	0.88 (0.70–1.10)	0.90 (0.74–1.10)	0.73 (0.57–0.92)
Perinatal						
Maternal smoking in pregnancy	1.44 (1.06–1.94)∗	1.41 (1.07–1.87)∗^,^†	1.38 (0.88–2.08)	1.17 (0.80–1.67)	1.18 (0.84–1.58)	1.11 (0.76–1.62)
Maternal alcohol use in pregnancy	1.57 (1.20–2.06)∗∗^,^††	1.57 (1.23–2.02)∗∗∗^,^††	1.32 (0.88–1.94)	1.37 (0.98–1.91)	1.35 (1.01–1.77)	1.12 (0.82–1.57)
Low birthweight (<2500 g)	1.02 (0.67–1.49)	1.05 (0.73–1.50)	1.47 (0.87–2.33)	1.06 (0.62–1.70)	1.04 (0.65–1.62)	1.81 (0.83–3.59)
Prematurity	1.00 (0.71–1.39)	1.05 (0.77–1.43)	0.65 (0.35–1.09)	1.07 (0.68–1.58)	1.09 (0.76–1.58)	0.57 (0.32–0.93)
Breastfeeding	1.28 (0.81–2.10)	1.28 (0.80–2.03)	1.25 (0.66–2.70)	1.38 (0.82–2.40)	1.26 (0.78–2.22)	1.24 (0.74–2.34)
Prenatal care ≥8 visits	0.98 (0.75–1.28)	1.02 (0.80–1.31)	0.93 (0.64–1.37)	1.06 (0.77–1.50)	1.06 (0.78–1.49)	0.97 (0.66–1.46)
Neonatal ICU admission	0.61 (0.38–0.93)∗	0.64 (0.42–0.98)∗	0.64 (0.31–1.17)	0.64 (0.38–1.03)	0.68 (0.41–1.07)	0.72 (0.31–1.37)
Eclampsia	0.99 (0.72–1.34)	1.03 (0.77–1.37)	0.91 (0.57–1.41)	0.87 (0.61–1.23)	0.90 (0.63–1.23)	0.87 (0.54–1.37)
Gestational diabetes	1.77 (1.04–2.87)∗	1.67 (1.05–2.65)∗	2.10 (1.07–3.71)∗	1.91 (1.01–3.28)	1.76 (0.95–2.86)	2.18 (1.13–3.83)
UTI in pregnancy	1.07 (0.79–1.44)	1.12 (0.84–1.48)	1.13 (0.72–1.70)	0.95 (0.66–1.34)	0.96 (0.67–1.29)	1.01 (0.64–1.53)
Other pregnancy complication	0.93 (0.52–1.55)	0.97 (0.58–1.60)	1.32 (0.62–2.44)	0.77 (0.40–1.33)	0.80 (0.43–1.33)	1.17 (0.43–2.36)
Sociodemographic						
Sex (female)	2.79 (2.16–3.62)∗∗∗^,^†††	2.61 (2.05–3.34)∗∗∗^,^†††	3.11 (2.14–4.61)∗∗∗^,^†††	**3.06 (2.28**–**4.17)**^a^	**2.88 (2.25**–**3.69)**^a^	**3.12 (2.19**–**4.49)**^a^
Race (white vs non-white)	1.02 (0.80–1.31)	0.99 (0.79–1.25)	1.15 (0.81–1.63)	1.17 (0.83–1.64)	1.14 (0.86–1.51)	1.22 (0.78–1.85)
Family						
Caregiver internalising disorder	1.48 (1.14–1.91)∗∗^,^†	1.45 (1.15–1.83)∗∗^,^††	1.30 (0.89–1.87)	1.02 (0.73–1.39)	1.03 (0.78–1.38)	0.95 (0.66–1.37)
Caregiver externalising disorder	0.47 (0.08–1.59)	0.51 (0.13–2.06)	1.76 (0.41–4.77)	0.03 (0.00–0.72)	0.03 (0.00–0.60)	0.15 (0.00–3.50)
Caregiver suicide attempt	2.24 (1.63–3.05)∗∗∗^,^†††	2.11 (1.60–2.79)∗∗∗^,^†††	1.54 (0.94–2.39)	2.07 (1.40–3.14)	1.88 (1.34–2.63)	1.32 (0.85–2.03)
Caregiver thought disorder	1.63 (1.13–2.29)∗∗^,^†	1.58 (1.15–2.18)∗∗^,^†	1.41 (0.83–2.27)	1.00 (0.60–1.56)	1.01 (0.67–1.57)	1.09 (0.65–1.82)
Adversity						
Childhood threat (z)	1.35 (1.21–1.52)∗∗∗^,^†††	1.31 (1.17–1.45)∗∗∗^,^†††	1.28 (1.09–1.51)∗∗^,^†	1.26 (1.08–1.47)	1.21 (1.06–1.38)	1.26 (1.05–1.51)
Childhood deprivation (z)	1.14 (1.01–1.29)∗	1.13 (1.00–1.27)∗	1.07 (0.90–1.28)	0.95 (0.81–1.12)	0.95 (0.83–1.09)	0.89 (0.75–1.07)
Cognitive						
IQ (z)	0.98 (0.87–1.11)	0.98 (0.88–1.10)	0.84 (0.71–1.00)	1.05 (0.88–1.22)	1.06 (0.92–1.23)	0.86 (0.69–1.08)
Executive function (z)	1.03 (0.91–1.17)	0.97 (0.86–1.09)	1.06 (0.89–1.27)	0.99 (0.84–1.20)	0.93 (0.79–1.09)	1.11 (0.86–1.45)
Clinical						
Any internalising disorder	1.41 (1.00–1.96)∗	1.44 (1.06–1.96)∗^,^†	1.15 (0.68–1.85)	0.92 (0.64–1.37)	0.96 (0.66–1.36)	0.83 (0.52–1.25)
Any externalising disorder	1.59 (1.15–2.17)∗∗^,^†	1.62 (1.22–2.16)∗∗∗^,^††	1.50 (0.94–2.29)	1.52 (1.05–2.15)	1.50 (1.07–2.08)	1.36 (0.91–1.96)
Recent DSH/suicidal talk	1.77 (1.00–2.99)∗	1.69 (1.04–2.75)∗	1.74 (0.78–3.34)	1.22 (0.63–2.21)	1.17 (0.64–2.03)	1.40 (0.62–2.53)

LR = logistic regression; Cox = Cox proportional hazards; QP = quasi-Poisson. OR = odds ratio; HR = hazard ratio; IRR = incidence rate ratio.

Single-predictor: ∗ p < 0.05, ∗∗ p < 0.01, ∗∗∗ p < 0.001 (uncorrected); † FDR < 0.05, †† FDR < 0.01, ††† FDR < 0.001.

Multivariable: bootstrap with 1000 iterations. Point estimates are means of bootstrapped log-coefficients (exponentiated); 95% CI from 2.5th–97.5th percentiles of log-scale distribution, exponentiated.

Bold indicates robust predictors. Genetic models adjusted for 10 ancestry principal components.

a Robust predictor (significant in ≥95% of bootstrap iterations).

Several factors showing single-predictor associations, including caregiver internalising disorders, caregiver thought disorder, childhood deprivation, and baseline internalising disorders, did not retain association with outcomes after mutual adjustment in multivariable models. Unweighted models (Table S13) yielded concordant estimates, with sex, childhood threat, caregiver suicide attempt, and externalising disorders remaining significant across logistic and Cox models.

**Fig. 1:**
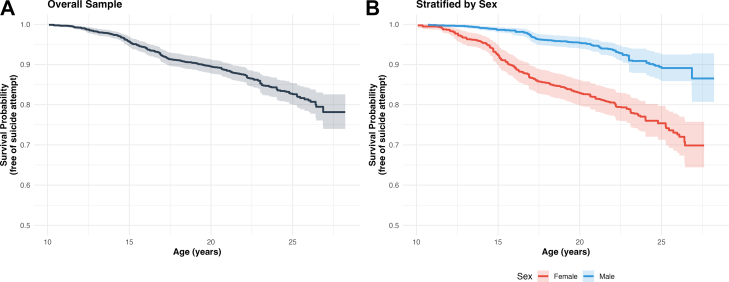
**Cumulative incidence of first suicide attempt from childhood to early adulthood**. Panel A shows the overall Kaplan–Meier cumulative incidence of first suicide attempt with age as the time scale (left truncation at age at baseline). Panel B presents cumulative incidence stratified by sex (female vs male), with 95% confidence intervals shown as shaded bands. Curves are weighted using CBPS weights to reflect the covariate distribution of the full screening sample (N = 9937). The log-rank test compared survival distributions between groups (p < 0.001 for sex). N = 2049 participants; 298 incident events. Vertical dashed lines indicate ages at which the 10% cumulative incidence threshold was reached for each group.

**Fig. 2:**
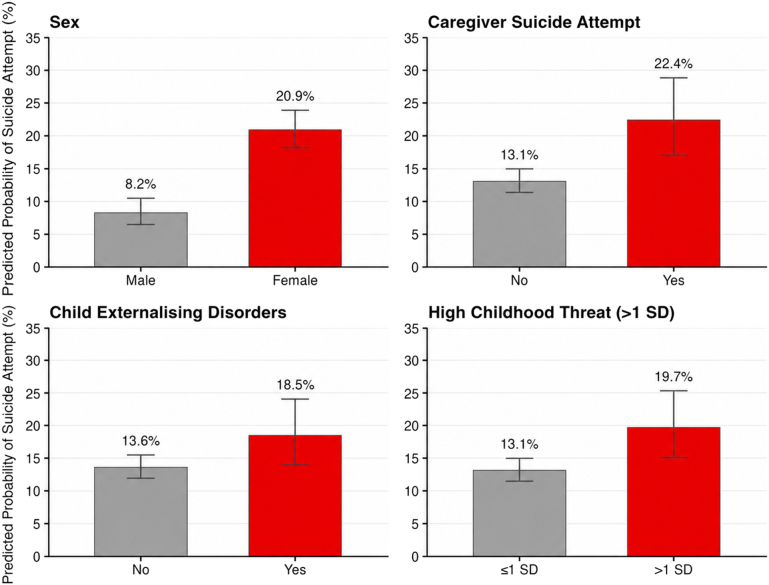
**Predicted probability of suicide attempt by key risk factor**. Predicted probabilities (with 95% confidence intervals) of any incident suicide attempt during the observed follow-up, derived from the multivariable weighted logistic regression model including all candidate risk factors simultaneously. The four panels display four prevention-relevant predictors that emerged as the most consistent findings across the multivariable bootstrap models (Table 2) and population attributable fraction analyses (Table 3): sex assigned at birth (female vs male) and childhood threat (continuous z-score, here dichotomised at >1 SD above the mean for visualisation, consistent with the threshold used in Table 3) were robustly associated with all three outcomes (occurrence, onset timing, and number of attempts); caregiver lifetime suicide attempt (yes vs no) and baseline childhood externalising disorders (yes vs no) were robustly associated with occurrence and earlier onset (Table 2). Probabilities represent average marginal predictions, conditional on the observed distribution of all other covariates in the model (race, deprivation, polygenic risk scores, perinatal exposures, IQ, executive function, internalising disorders, recent self-harm talk, caregiver internalising disorder, caregiver thought disorder, and caregiver externalising disorder). Confidence intervals were obtained from 1000 bootstrap iterations. The corresponding combination-level predicted probabilities, ranging from 4.3% (males without caregiver suicide attempt, externalising disorders, or high threat exposure) to 60.0% (females with all three risk factors present), are reported in the Results section.

Kaplan–Meier survival curves (Fig. 1) illustrated marked differences in onset timing. Panel A shows cumulative incidence rising from 0% at age 10 to 5.7% by age 16, 10.5% by age 20, and 18.9% by age 26. Panel B, stratified by sex, reveals that females had substantially steeper trajectories than males (log-rank p < 0.001). Estimated cumulative incidence at age 30, extrapolated beyond the mean Wave 3 follow-up age (23.2 years; SD 1.9) and therefore interpreted with caution, was 30.1% for females vs 13.5% for males, 29.3% for those with caregiver suicide attempt vs 20.6% without, 28.9% for high childhood threat vs 15.3% for low threat, and 24.3% for those with baseline externalising disorders vs 21.0% without. Notably, females reached the 10% cumulative incidence threshold 8.8 years earlier than males (age 15.7 vs 24.5 years), offspring of suicide attempters reached this threshold 6.5 years earlier (age 14.7 vs 21.2 years), those with high threat exposure reached it 6.1 years earlier (age 16.9 vs 23.0 years), and those with externalising disorders reached it 4.6 years earlier (age 16.1 vs 20.7 years). Additional stratified curves appear in Figures S3–S6. Fig. 2 illustrates predicted probabilities across risk factor combinations, ranging from 4.3% (males without caregiver suicide attempt, externalising disorders, or high threat exposure) to 60.0% (females with all three risk factors present).

### Population attributable fractions (PAF)

Population attributable fractions quantified the potential public health impact of each risk factor (Table 3). Caregiver suicide attempt had the highest PAF (14.1%, 95% CI 7.3–21.4%), followed by caregiver internalising disorders (11.6%, 95% CI 3.4–20.2%), high childhood threat exposure (>1 SD; 11.5%, 95% CI 5.5–19.1%), and child externalising disorders (10.2%, 95% CI 3.6–16.8%). Child internalising disorders (4.5%, 95% CI −0.4 to 10.1%) and recent self-harm talk (2.9%, 95% CI −0.0 to 6.8%) had non-significant PAFs.

**Table 3:** Population attributable fractions for main risk factors.

Risk factor	Population prevalence	OR	PAF (95% CI)	
Caregiver Suicide Attempt	12.2%	2.35	14.1% (7.3%–21.4%)	a
Caregiver Internalising Disorder	29.0%	1.45	11.6% (3.4%–20.2%)	a
High Childhood Threat (>1 SD)	16.5%	1.79	11.5% (5.5%–19.1%)	a
Child Externalising Disorder	13.8%	1.82	10.2% (3.6%–16.8%)	a
Child Internalising Disorder	12.7%	1.37	4.5% (−0.4% to 10.1%)	
Recent Self-Harm Talk	3.7%	1.80	2.9% (−0.0% to 6.8%)	
Caregiver Externalising Disorder	1.3%	0.43	N/A (protective)	

PAF = population attributable fraction calculated using Levin's formula: PAF = P_pop(OR−1)/[1 + P_pop(OR−1)], where P_pop is the population prevalence of exposure (column 2). OR is adjusted for sex and age. Bootstrap 95% CI (1000 iterations).

Risk factors ordered by PAF magnitude.

PAF is a population-impact metric for risk factors (OR>1) and is not defined for protective factors (OR<1); for caregiver externalising disorder, the corresponding metric is the prevented fraction, not reported here.

Because the cumulative incidence of suicide attempts in this cohort (15.0%) is not rare, OR-based Levin PAFs may slightly overestimate risk-ratio-based attributable fractions; PAFs should be interpreted as approximate population-impact metrics rather than directly preventable proportions.

a Indicates that the bootstrap 95% CI for the PAF excludes zero. High childhood threat exposure was defined as >1 SD above the mean.

Decomposition of childhood threat into specific components by informant (Table S14) showed that, among child-reported exposures, bullying had the largest PAF (23.8%), followed by emotional abuse (8.6%) and physical abuse (7.0%); among parent-reported exposures, emotional abuse (21.8%), physical abuse (17.4%), and bullying (14.6%) contributed the largest fractions. These findings highlight bullying and intrafamilial abuse as high-impact prevention targets due to their combination of high prevalence and moderate-to-strong associations with suicide attempts.

Sequential model-building (Table S8) showed that sociodemographic factors explained the largest initial share of variance (Nagelkerke pseudo-R^2^ = 0.093), followed by adversity (ΔR^2^ = 0.044), the genetic block (ΔR^2^ = 0.052), and perinatal factors (ΔR^2^ = 0.025). The full model explained 24.3% of outcome variance. Comparison of baseline characteristics between completers (n = 2060) and non-completers (n = 451) revealed differential attrition by sex (p < 0.001), deprivation (p = 0.004), and IQ (p = 0.018); other variables did not differ significantly (Table S15). Sensitivity analyses combining CBPS selection weights with inverse probability of attrition weights (IPAW) for the full analytic sample (N = 2060) yielded substantively unchanged estimates across all predictors (all OR changes <4%; Table S6), indicating that differential attrition did not materially bias findings. The global Schoenfeld residual test was significant (χ^2^ = 171.2, df = 39, p < 0.001; Table S16), with several predictors showing non-proportionality, including sex, childhood threat, and caregiver suicide attempt. The direction of association was consistent for all predictors. Restricting Cox models to 229 attempters with confirmed C-SSRS dates (Cox sample N = 1980) yielded substantively unchanged hazard ratios (Table S3).

### Supplemental analyses

Threat component analyses by informant source (Table S17) revealed child-reported bullying as a key threat component that retained association with suicide attempts in multivariable models including all components simultaneously (OR 1.73, 95% CI 1.22–2.45; HR 1.75, 95% CI 1.24–2.47); parent-reported physical abuse also retained association across all three models (OR 1.96, 95% CI 1.24–3.10; HR 1.86, 95% CI 1.18–2.95; IRR 1.87, 95% CI 1.11–3.17). Lethality analyses comparing 91 high-lethality attempters to 1751 non-attempters (Table S18) identified a broader set of risk factors for medically serious attempts. Female sex (OR 3.69, 95% CI 1.85–7.43; HR 4.80, 95% CI 2.77–9.18; IRR 3.16, 95% CI 1.74–7.76) and PRS-depression (OR 2.21, 95% CI 1.09–4.43; HR 1.98, 95% CI 1.13–3.60; IRR 2.68, 95% CI 1.43–4.97) retained association across all three multivariable models. Childhood threat was associated with onset after mutual adjustment (HR 1.40, 95% CI 1.09–1.80), number of high-lethality attempts (IRR 1.34, 95% CI 1.03–1.90), and occurrence (OR 1.41, 95% CI 1.01–1.94). Among perinatal factors, maternal alcohol use in pregnancy was associated with earlier onset of high-lethality attempts after mutual adjustment (HR 1.92, 95% CI 1.12–3.26), with a borderline association for occurrence (OR 1.79, 95% CI 1.00–3.08), and having ≥8 prenatal care visits was associated with occurrence of high-lethality attempts (OR 2.00, 95% CI 1.06–4.05). PRS-suicide death showed an inverse association with the number of high-lethality attempts (IRR 0.66, 95% CI 0.43–0.98). Notably, caregiver suicide attempts and baseline externalising disorders, associated with overall attempts in multivariable models, were not associated with high-lethality attempts specifically.

Within-attempter analysis (Table S19) revealed that PRS-depression (OR 1.61, 95% CI 1.29–2.05), PRS-anxiety (OR 1.54, 95% CI 1.24–1.94), and UTI in pregnancy (OR 2.07, 95% CI 1.40–3.05) differentiated lethality. Female sex was not significant (OR 1.01), and caregiver suicide attempt showed an inverse association (OR 0.56, 95% CI 0.36–0.86). When the five PRS were replaced by PC1 (63.6% of variance; Table S7), higher genetic liability was associated with incidence (OR 1.28, 95% CI 1.08–1.53) and onset (HR 1.26, 95% CI 1.01–1.56) but not frequency (IRR 1.01, 95% CI 0.84–1.23). In the ideation-restricted analysis (Table S20), among the primary predictors of interest, female sex (OR 1.75), caregiver suicide attempt (OR 1.84), externalising disorders (OR 1.84), and gestational diabetes (OR 4.78) remained significant. Childhood threat was not (OR 1.03, 95% CI 0.85–1.25), suggesting its association with suicide attempts may largely reflect shared ideation risk rather than a transition-specific effect. Additional perinatal predictors (maternal alcohol use in pregnancy OR 1.58, breastfeeding OR 2.06, neonatal ICU admission OR 0.56) reached significance in this restricted analysis and are reported in Table S20 as exploratory findings; given the cross-sectional ascertainment of ideation and attempt at Wave 3, these results should be interpreted with caution and do not establish prospective ideation-to-action pathways. PAFs with fully adjusted ORs are reported in Table S9 (Panel A) and were attenuated relative to minimally adjusted estimates, especially for childhood threat (11.5% → 5.6%) and caregiver suicide attempt (14.1% → 10.7%); fully adjusted PAFs were undefined for caregiver internalising and child internalising disorders, whose adjusted ORs fell below 1.00. Threat PAFs were stable across alternative thresholds (>0.5 SD: 13.3%; >1.0 SD: 11.5%; >1.5 SD: 9.3%; Table S9, Panel B).

## Discussion

In this 15-year prospective cohort of Brazilian youth, childhood factors spanning genetic, environmental, and clinical domains showed robust associations with later suicide attempts, yet even after integrating seven risk factor domains, predictive discrimination remained modest (mean AUC 0.665), and nonlinear machine learning classifiers did not outperform logistic regression. Although this exceeds the weighted AUC of 0.61 reported in a meta-analysis of 50 years of suicide prediction research,^49^ it remains within the “prediction ceiling” consistently observed in the field and insufficient for individual-level clinical decision-making. This ceiling likely reflects that suicidal behaviour is driven by proximal, rapidly fluctuating processes that static baseline risk factors cannot fully capture, reinforcing the importance of population-level prevention over individual risk stratification with distal factors alone. Several factors likely contribute to this ceiling. First, baseline predictors are distal and trait-like, whereas suicide attempts are acute, state-dependent events triggered by proximal processes (interpersonal crises, substance use, sleep disruption, access to means) that change rapidly and are not captured by childhood assessments. Second, the outcome “any lifetime suicide attempt” aggregates heterogeneous trajectories (first vs repeat attempts, low-vs high-lethality events), each potentially driven by partially distinct mechanisms, diluting discriminative performance. Third, as our ideation-restricted analysis suggests, some predictors (e.g., childhood threat) may primarily increase ideation prevalence rather than specifically facilitate the behavioural transition to action, meaning that models conflating shared ideation risk with transition-specific risk will have inherently limited specificity. Fourth, strong etiologic associations do not automatically translate into high predictive discrimination; an odds ratio of 3.0 for a moderately prevalent exposure yields only a modest improvement in AUC.^50^ Together, these considerations argue against relying primarily on clinical screening of high-risk individuals based on distal childhood factors and instead support a shift toward structural, population-level interventions—such as anti-bullying policies, family-based programmes for offspring of caregivers with suicidal behaviour history, and early intervention for childhood externalising disorders—whose population impact does not depend on accurate individual-level prediction.

The 15.0% lifetime prevalence observed in our sample falls at the upper end of global estimates in children and adolescents (ranging from 4.6% to 16.9%),^51^ likely reflecting the extended follow-up into young adulthood and comprehensive assessment methods capturing attempts that might be missed in briefer surveys.^52^^,^^53^ The threefold increased risk among females (OR 3.06; HR 2.88; IRR 3.12) aligns with global epidemiological patterns showing higher rates of suicide attempts in adolescent girls despite higher suicide mortality in males.^54^ A meta-analysis of longitudinal studies found that females have approximately twice the risk of suicide attempts compared with males (OR 1.96, 95% CI 1.54–2.50)^55^—lower than observed in our sample (OR 3.06). This sex differential, which emerged around age 12 and widened through adolescence in our survival analyses, likely reflects the confluence of biological factors (pubertal hormonal changes), psychological factors (higher rates of internalising disorders in females), and social factors (differential exposure to interpersonal stressors and sexual violence).^54^^,^^56^ Females reached the 10% incidence threshold 8.8 years earlier than males (age 15.7 vs 24.5 years). The larger difference observed in our sample compared with prior meta-analytic estimates^55^ may be explained by contextual factors specific to a middle-income Latin American setting, where rates of intimate partner violence and gender-based violence are among the highest globally and have been shown to mediate the association between sex and suicidal ideation in Brazil,^57^ with violence against women consistently identified as one of the strongest risk factors for suicide attempts across low- and middle-income countries.^58^ Future analyses of this cohort should examine whether risk factor associations differ by sex through interaction analyses and sex-stratified models, which may reveal sex-specific prevention targets.

Three core risk factor domains showed robust, mutually adjusted associations with suicide attempts, each likely reflecting partially distinct pathways. First, childhood threat exposure, particularly peer victimisation and physical abuse (Table S17), was associated with all three outcomes (Table 2), consistent with theoretical models emphasising interpersonal trauma and social defeat in suicidal behaviour.^19^^,^^59^ As noted below, however, the ideation-restricted analysis suggests that part of this association may be mediated through suicidal ideation rather than reflecting a transition-specific effect on suicidal behaviour. Second, caregiver suicide attempt history was associated with incidence and earlier onset after adjustment for genetic risk scores and environmental factors, suggesting transmission mechanisms beyond shared genetic liability, including modelling of suicidal behaviour, shared family environments characterised by psychopathology and dysfunction, and gene–environment correlations.^60^, ^61^, ^62^ In contrast, caregiver internalising disorders lost significance in mutually adjusted models, indicating that the specific history of suicidal behaviour, rather than general parental psychopathology, carries prognostic importance, consistent with family studies demonstrating the specificity of familial transmission.^63^ Third, childhood externalising disorders were associated with incidence and earlier onset but not with the number of attempts, suggesting that impulsivity and emotion dysregulation may be particularly relevant to the transition to a first attempt rather than to the perpetuation of suicidal behaviour.^59^^,^^64^, ^65^, ^66^ Importantly, these associations represent statistical adjustments for co-occurring risk factors and should not be equated with causal independence; unmeasured confounders, including neighbourhood environment, ongoing adversity beyond baseline, and peer influences, remain plausible alternative explanations. The convergence of interpersonal, familial, and behavioural pathways after mutual adjustment nonetheless supports multi-level prevention approaches targeting each domain.

The ideation-restricted analysis provides preliminary evidence regarding risk factor specificity. Caregiver suicide attempt, externalising disorders, and female sex remained associated with attempts among ideators, consistent with ideation-to-action frameworks.^18^^,^^19^ Childhood threat was not associated with attempts among ideators (OR 1.03, 95% CI 0.85–1.24), suggesting it may primarily increase ideation prevalence rather than facilitate the behavioural transition. This pattern has implications for prevention design: threat-reduction strategies may reduce ideation at the population level, while interventions targeting behavioural disinhibition and family transmission may more specifically prevent the transition from ideation to action.

Among the specific threat components, child-reported bullying and parent-reported physical abuse were the exposures that remained associated with suicide attempts when all threat types were entered simultaneously (Table S17), underscoring the importance for interpersonal violence (peer victimisation and intrafamilial abuse) in adolescent suicidal behaviour.^5^ Given that bullying is among the most prevalent modifiable risk factors for mental health disorders in youth^67^ and that our cohort was recruited from state-funded schools, these findings have direct relevance to Brazil's public education system. The combination of high prevalence and consistent association of bullying with suicide attempts (Table S17) suggests that effective implementation of evidence-based anti-bullying programmes across the state-funded school network could contribute to meaningful reductions in adolescent suicide attempts at the population level. Importantly, the value of targeting bullying lies in its combination of high prevalence, moderate effect size, and actionable intervention context—not in its ability to identify specific individuals who will attempt suicide. This distinction between population-attributable impact and individual-level prediction is central to our findings. We note, however, that while meta-analyses demonstrate that anti-bullying programmes reduce bullying perpetration and victimisation,^68^^,^^69^ direct evidence that such programmes reduce suicide attempts specifically remains limited and represents an important area for future intervention research.

Several secondary findings warrant cautious interpretation as hypothesis-generating signals. Polygenic risk scores showed outcome-specific rather than global associations: PRS-depression was associated with the number of attempts and, in lethality analyses, consistently predicted medically serious attempts across all three models, suggesting that genetic liability to depression may influence the persistence and severity of suicidal behaviour more than its initiation.^70^^,^^71^ PRS-suicide death showed an unexpected inverse association with attempt frequency, possibly reflecting survivor bias or genetically distinct architectures underlying fatal vs non-fatal suicidal behaviour.^4^ The composite genetic score (PC1) confirmed that overall polygenic risk was associated with incidence and onset even though individual PRS showed limited independent effects due to high inter-correlations; the PRS block collectively explained 5.2% of additional variance beyond environmental predictors. Individual PRS coefficients represent conditional effects; a non-significant coefficient does not imply that the underlying genetic liability is unimportant, but rather that its signal overlaps with adjusted scores. Perinatal factors similarly showed outcome-specific patterns consistent with the developmental origins framework^9^^,^^72^, ^73^, ^74^: maternal alcohol use in pregnancy was associated with earlier onset,^75^^,^^76^ gestational diabetes with attempt frequency,^77^ and a marker of high-risk pregnancy (≥8 prenatal care visits) with high-lethality attempts specifically; however, these retrospectively assessed exposures are susceptible to differential recall bias and confounding by indication, and should not be interpreted as directly causal. In lethality analyses, PRS-depression and childhood threat were the most consistent predictors of medically serious attempts,^78^^,^^79^ while within-attempter comparisons revealed that genetic scores differentiated high-from low-lethality attempters but female sex did not (OR 1.01), suggesting that the marked sex disparity operates through propensity for suicidal behaviour rather than method severity. Similarly, caregiver suicide attempt was inversely associated with lethality among attempters (OR 0.56), suggesting that familial transmission increases propensity for attempting without necessarily increasing medical severity.^19^ These outcome-specific and model-specific associations emerged predominantly in supplementary analyses and require replication before causal or clinical interpretation.

This study has several notable strengths. The Brazilian High-Risk Cohort is one of the few prospective cohorts from a low- or middle-income country with approximately 15 years of follow-up and 82% retention, providing rare longitudinal evidence on suicide attempts from childhood into early adulthood in an underrepresented setting. Risk factors spanning genetic, perinatal, sociodemographic, family, adversity, cognitive, and clinical domains were assessed at baseline and analysed simultaneously, enabling examination of mutually adjusted associations across a developmental framework rarely integrated in prior work. Three complementary analytical approaches—logistic regression, Cox proportional hazards, and quasi-Poisson regression—captured distinct risk dimensions (cumulative incidence, onset timing, and attempt frequency). Importantly, incident-attempt analyses excluded participants whose first suicide attempt occurred before baseline, ensuring that the predictors examined here genuinely precede a first attempt and supporting a developmental interpretation of risk. Use of covariate-balancing propensity score weights, inverse probability of attrition weights, multiple imputation, bootstrap inference, and a series of pre-specified sensitivity analyses (including ideation-restricted, within-attempter, and high-lethality comparisons) provides additional robustness to the main findings.

### Limitations

Some limitations warrant consideration. First, suicide attempts were assessed retrospectively at follow-up, introducing potential recall bias and precluding precise temporal ordering of risk factors and outcomes; however, we used DAWBA assessments at intermediate waves to anchor timing of first attempts when exact dates were unavailable, and our survival analyses employed age as the time scale rather than time-since-baseline, providing more epidemiologically appropriate estimates. Second, our multivariable models do not account for causal pathways between risk factors, potentially underestimating the importance of upstream factors whose effects operate through mediators included in the same model; the use of three complementary analytical approaches partially mitigates this by capturing distinct dimensions of risk. Third, polygenic risk scores derived from predominantly European-ancestry GWAS have documented reduced predictive validity in admixed populations^80^; while adjustment for ten genetic principal components addresses population stratification, it does not fully compensate for reduced PRS portability across ancestries. The PRS effects reported here likely represent lower-bound estimates of the true genetic contribution, and our genetic findings should be interpreted as preliminary until GWAS from Latin American and admixed populations become available. Nevertheless, this is among the first studies to integrate polygenic scores with environmental and clinical predictors of suicide attempts in a middle-income country. Fourth, our findings from an urban Brazilian cohort may not generalise to other cultural contexts or rural populations. Fifth, the high-risk enrichment procedure selected participants based on psychiatric symptoms and family history, potentially underrepresenting individuals whose risk arises from other pathways (e.g., perinatal factors alone). The inclusion of 958 randomly selected participants (38% of the analytic sample) partially mitigates this concern, and CBPS weights were applied to recover the covariate distribution of the full screening sample (N = 9937). Sixth, PAF estimates should be interpreted with caution: they assume causal relationships, are not additive when exposures are correlated, and do not account for potential mediation among risk factors. Because the cumulative incidence of suicide attempts in this cohort (15.0%) is not rare, OR-based Levin PAFs may slightly overestimate risk-ratio-based attributable fractions; PAFs are therefore interpreted as approximate population-impact metrics rather than directly preventable proportions. Future research with temporally ordered measurements and risk-ratio-based estimation (e.g., marginal standardisation or g-computation) could refine these interrelationships. Seventh, although perinatal factors were assessed using both medical records and clinical interviews with the main caregiver, recall bias for interview-based measures should be considered. Eighth, some threat indicators were available only from parental reports, which may underestimate exposure given that parents are often unaware of their children's experiences; however, for variables assessed by both informants, child-reported bullying and parent-reported physical abuse retained association with suicide attempts after mutual adjustment in threat-component models (Table S17), suggesting that both the child's perception of peer victimisation and parental reports of physical violence carry prognostic value above and beyond other co-occurring threat exposures. Ninth, several predictors showed non-proportional hazards (Table S16), meaning their HRs vary over the age range and represent weighted average effects. The concordance between Cox and logistic regression findings mitigates concern that these violations substantially affected conclusions for predictors identified as significant. However, a consequence of non-proportionality is that significant time-dependent effects may remain undetected when averaged across the full follow-up interval: predictors that did not reach significance in our models may nonetheless exert significant effects restricted to specific developmental windows—for example, childhood only or early adolescence only. Significant associations reported here using logistic regression or Cox models should therefore be considered robust, but apparent null findings should not be interpreted as evidence of no effect, as developmentally circumscribed effects could plausibly be present and warrant investigation in future analyses with time-stratified or time-varying coefficient models. Differences between models may also reflect differential handling of loss to follow-up rather than temporal dynamics of risk. Tenth, given the large inferential space, some findings observed in only one model or with borderline CIs should be interpreted as hypothesis-generating. Eleventh, the dichotomisation of race/ethnicity into white vs non-white obscures heterogeneity, particularly given the predominantly mixed-race/Pardo (31.1%) and Black/African-Brazilian (14.2%) composition of the non-white category. Additionally, although temporal ordering supports prospective interpretation, observational associations do not establish causality; unmeasured confounders such as neighbourhood environment and ongoing adversity beyond baseline remain plausible, and a PRS sensitivity analysis restricted to a higher European-ancestry subgroup was not feasible given the continuously admixed nature of the Brazilian population. Twelfth, caregivers of children already exhibiting psychiatric symptoms at baseline might disproportionately recall or report earlier adversities and perinatal complications, potentially inflating associations between these retrospectively assessed risk factors and offspring outcomes. Finally, participants had a mean age of 23 years at last assessment and have not yet passed through the full period of risk for suicide attempts, particularly males whose cumulative incidence continued to rise through the mid-twenties in our survival analyses; some current non-attempters may convert in subsequent years, suggesting that our effect sizes may represent conservative estimates.

### Conclusion

These findings are consistent with a multi-level approach to suicide prevention that extends beyond the health sector. The consistent associations of female sex and childhood threat with cumulative incidence, earlier onset, and number of attempts are relevant to cross-sector efforts to prevent childhood trauma across the settings in which children live, learn, and interact, recognising that childhood threat may operate in part by increasing suicidal ideation rather than exclusively by facilitating the behavioural transition to action. The association with caregiver suicide attempts is consistent with parental mental health and family-level risk being relevant components of prevention, with potential downstream benefits for offspring. The identification of childhood externalising disorders as factors associated with cumulative incidence and earlier onset indicates that suicide risk should be assessed in youth presenting with behavioural problems, not only internalising symptoms. However, given the modest predictive performance of current models, including ours, and the limited evidence for universal screening efficacy, particularly in resource-constrained settings, these findings support a complementary approach: population-level prevention targeting high-impact modifiable exposures such as bullying and childhood adversity, alongside clinical vigilance for suicide risk in youth who present with known risk factors such as externalising disorders, adversity exposure, or family history of suicidal behaviour. At the population level, the large attributable fraction for bullying supports prioritisation of school-based prevention strategies. Although biological factors, including PRS-depression and gestational diabetes, were associated with the number and medically serious attempts, overall predictive performance remained modest, and nonlinear models did not improve discrimination, consistent with the persistent limits of current individual-level prediction and arguing for continued methodological refinement while prioritising broad prevention strategies and clinical judgment over risk stratification alone. Findings observed consistently across all three analytical approaches and confirmed in sensitivity analyses represent the most robust associations, whereas results emerging in a single model, with borderline confidence intervals, or only in supplementary analyses should be interpreted as hypothesis-generating.

## Contributors

RFD had unrestricted access to all study data and verified the underlying data reported in the manuscript. RFD led conceptualisation, methodology, formal analysis, data curation, software, validation, visualisation, project administration, and writing of the original draft. DML contributed to formal analysis, methodology, software, and validation, with particular responsibility for the bootstrap multivariable inference framework and machine-learning benchmarking. DML reviewed and edited the manuscript. GAS had unrestricted access to all study data and verified the underlying data reported in the manuscript. GAS contributed to conceptualisation, methodology, formal analysis, supervision, funding acquisition for the BHRC, investigation, and resources, and reviewed and edited the manuscript. LTI, MLS, SB, PMP, CC, and JLS contributed to conceptualisation, investigation, data curation, methodology, and resources, including the design of the Brazilian High-Risk Cohort Study, participant recruitment and retention, and the genetic, perinatal, and clinical data collection and processing pipelines underpinning the analyses reported here. LTI, MLS, SB, PMP, CC, and JLS reviewed and edited the manuscript. ECM and LAR contributed to conceptualisation, investigation, methodology, supervision, project administration, funding acquisition for the BHRC, and resources as principal investigators of the Brazilian High-Risk Cohort Study from its inception. ECM and LAR reviewed and edited the manuscript. RU, PS, CJB, and HPB contributed to methodology, validation, supervision, and interpretation of findings, with specific clinical and methodological expertise in suicide research, neuroimaging, polygenic risk score interpretation, and developmental psychopathology. RU, PS, CJB, and HPB reviewed and edited the manuscript and provided critical revisions for important intellectual content. All authors reviewed and approved the final version of the manuscript and agree to be accountable for all aspects of the work. RFD and GAS had final responsibility for the decision to submit for publication. RFD and GAS, the latter from the academic team that initiated and continues to lead the BHRC, jointly accessed and verified the underlying data.

## Data sharing statement

Data are available upon reasonable request. For data access requests, please contact the corresponding author at damianorf@usp.br.

## Declaration of generative AI and AI-assisted technologies

During the preparation of this work, the authors used Claude (Anthropic) for grammar and language refinement of selected manuscript sections. The tool was not used to generate scientific content, interpret data, draft results, or produce references. After using this tool, the authors reviewed and edited the content as needed and take full responsibility for the content of the publication.

## Declaration of interests

RFD received honoraria for lectures and educational material from Johnson and Johnson and Viatris. RU has received grants unrelated to the present work from the Canadian Institutes of Health Research, the Ontario Brain Institute, and Janssen Canada, and editorial fees from the American Medical Association. HPB has consulted to Eli Lilly, Boehringer Ingelheim, and BioHaven. PMP received honoraria for lectures, presentations, and speakers' bureaus from Germed Pharma. LAR has received grants unrelated to the present work from the National Council for Scientific and Technological Development (CNPq) and the United States National Institutes of Health (R01MH120482); royalties from Artmed and Oxford Press; consulting fees from Adium, Apsen, Medice, Novartis/Sandoz, and Shire/Takeda; honoraria for lectures, presentations, speakers' bureaus, manuscript writing, or educational events from Abdi Ibrahim, Abbott, Aché, Adium, Apsen, Bial, Medice, Novartis/Sandoz, Pfizer/Upjohn/Viatris, and Shire/Takeda; support for attending meetings and/or travel from the Stavros Niarchos Foundation; has participated on Data Safety Monitoring or Advisory Boards for Adium, Apsen, EMS, Libbs, Medice, Novartis/Sandoz, and Shire/Takeda; and holds a leadership role in the International Association of Child and Adolescent Psychiatry and Allied Disciplines.
