# Supplementary material for: Developmental predictors of suicide attempts from childhood to early adulthood: a 15-year prospective cohort study
Source: Lancet Reg Health Am. 2026 Jun 25;61:101531. doi: 10.1016/j.lana.2026.101531 (PMC13320554; doi:10.1016/j.lana.2026.101531)
Supplement: Supplementary Methods [file mmc1.docx]

Supplementary Methods

***Survey Weights***

Covariate-balancing propensity score (CBPS) weights^1^ were estimated for the Wave 3 analytic sample to recover the covariate distribution of the full screening sample (N=9,937). The weighting model included all variables used in the original oversampling procedure: state, child age, child sex, number of biological and half-siblings, and family liability indices for attention-deficit/hyperactivity disorder, anxiety disorders, obsessive-compulsive disorder, psychotic disorders, and learning disorders from both the screened child and non-screened siblings. This approach yields effect estimates generalisable to the target population of children enrolled in public schools, rather than reflecting the enriched high-risk cohort, and also adjusts for differences related to differential selection. Weight distribution diagnostics, effective sample size, standardised mean differences before and after weighting, and sensitivity to weight trimming at the 1st/99th and 5th/95th percentiles are reported in Table S4.

***Inverse Probability of Attrition Weights (IPAW)***

To address potential bias from differential loss to follow-up, we estimated inverse probability of attrition weights using Wave 3 completion status in the original deeply phenotyped cohort (451 non-completers among N=2,511). A logistic model regressed Wave 3 completion on baseline covariates (age, sex, race, childhood threat, childhood deprivation, baseline psychopathology, IQ, executive function, caregiver characteristics, and PRS). Combined CBPS × IPAW weights (final weight = CBPS × IPAW) were applied to the incident-risk analytic sample (N=2,060). Main models were re-run with the combined weights as a sensitivity analysis (Table S6). All OR changes were <4%, indicating that differential attrition did not materially bias findings.

***Missing Data Handling***

Multiple imputation using chained equations (MICE) addressed missing risk factor data. We generated 5 imputed datasets with 10 iterations each. Predictive mean matching was used for continuous variables and logistic regression for binary variables. Outcome variables (suicide attempt occurrence and count) were not imputed but were included as auxiliary variables in the imputation model to improve imputation quality under the missing-at-random assumption. Results were pooled across imputed datasets using Rubin’s rules. Per-variable missingness is reported in Table S5.

***Single-Predictor Analyses***

Each risk factor was examined separately using three complementary model types: **logistic regression** for any incident suicide attempt (binary outcome) using survey-weighted generalised linear models with quasi-binomial family and CBPS weights; **Cox proportional hazards regression** for time to first suicide attempt, using age as the time scale with left truncation (delayed entry) to account for varying ages at study enrolment—sampling weights were incorporated via robust variance estimation with clustering by participant ID; the date of the first attempt was obtained from the C-SSRS when available, and for participants reporting incident attempts without specific dates, timing was imputed from the earliest wave with endorsed deliberate self-harm on the DAWBA; participants whose first attempt occurred before baseline assessment were excluded; and **quasi-Poisson regression** for the number of incident attempts using survey-weighted generalised linear models, with the quasi-Poisson family to account for overdispersion in count data. The estimated dispersion parameter was φ = 10·4, confirming substantial overdispersion. Quasi-Poisson was preferred over negative binomial regression because it accommodates overdispersion without imposing a specific mean–variance relationship and is directly compatible with survey weighting via the quasipoisson family in R.

For single-predictor models, false discovery rate (FDR) correction using the Benjamini-Hochberg procedure was applied to control for multiple comparisons across all predictors within each model type. Statistical significance was indicated at both uncorrected (p<0·05) and FDR-corrected (q<0·05) thresholds.

***Bootstrap Multivariable Inference***

Rather than employing variable selection techniques (e.g., elastic-net, stepwise), which can introduce post-selection bias and underestimate standard errors, we adopted a bootstrap multivariable approach that includes all candidate risk factors simultaneously. This methodology follows established statistical principles for valid inference^2,3^.

**Procedure:** For each of N=1,000 bootstrap iterations, participants were resampled with replacement from the analytic sample (maintaining the imputation structure by cycling through imputed datasets); the full multivariable model including all risk factors simultaneously was fit; and exponentiated coefficients (OR, HR, or IRR) were extracted for each risk factor.

**Summary statistics:** Effect estimates were the mean of bootstrapped log-coefficients across 1,000 iterations, exponentiated for interpretation. 95% confidence intervals were derived from the 2·5th and 97·5th percentiles of the log-scale bootstrap distribution, exponentiated. Statistical significance was indicated when the confidence interval excluded 1·00.

The decision to use the bootstrap mean rather than the median was based on assessment of bootstrap distribution skewness on the log scale (Table S1, Panel B). Mean |skewness| was 0·21 across all three models, with all predictors except caregiver externalising disorder showing |skewness| < 0·5, supporting the use of the mean as the point estimate. Convergence rates were 1,000/1,000 across all three model types (Table S1, Panel A). Monte Carlo standard errors of CI endpoints were small (<0·005 for all predictors), indicating stable CI estimation.

**CBPS weights were held fixed across bootstrap resamples;** this is a practical compromise that may slightly underestimate variance and is acknowledged in the Limitations.

**Model specifications:** Logistic regression: quasi-binomial family with CBPS weights. Cox regression: robust variance with CBPS weights and clustering by participant ID. Quasi-Poisson: quasi-Poisson family with CBPS weights. For models including polygenic risk scores, 10 principal components of genetic ancestry were included as covariates to control for population stratification.

***Unweighted Sensitivity Analysis***

To verify that findings were not artefacts of the CBPS weighting procedure, we repeated the full bootstrap multivariable analysis without survey weights. Concordance between weighted and unweighted results was assessed by comparing effect estimates and significance patterns (Table S13).

***Collinearity Diagnostics***

Prior to multivariable modelling, we assessed multicollinearity among risk factors using **generalised variance inflation factors (GVIF)**, calculated for all risk factors using car::vif in R; for categorical predictors with df>1, GVIF^(1/2Df) was reported. Values >5 would indicate concerning collinearity. We also examined a **Spearman correlation matrix** of pairwise correlations among all continuous and binary risk factors. Results indicated acceptable collinearity levels: all GVIF values were below 5 (only PRS values were >2; Table S2). Spearman correlations were generally low, with a median r=0·05 and 93·7% of pairwise correlations showing |r|<0·3 (Figure S2). Higher correlations (|r|>0·5) were observed exclusively among polygenic risk scores (range 0·61–0·75), reflecting established genetic pleiotropy across psychiatric phenotypes; these scores were retained as separate risk factors given their distinct theoretical constructs.

***Sequential Model-Building***

To quantify the incremental contribution of each risk factor domain, we fit sequential weighted logistic models adding domains cumulatively in temporal order: sociodemographic → adversity → family → clinical → perinatal → cognitive → genetic. Nagelkerke pseudo-R² and its change (ΔR²) at each step are reported in Table S8. Likelihood ratio tests were not used because they are not valid under survey weighting; pseudo-R² differences are descriptive rather than inferential.

***Machine Learning Comparison***

To benchmark the predictive performance of our bootstrap logistic regression approach, we compared discrimination against four machine learning classifiers: **Elastic Net** (regularised logistic regression with tuned mixing parameter and lambda); **Random Forest** (500 trees with tuned mtry); **XGBoost** (gradient boosting with early stopping and tuned learning rate, max depth, and number of rounds); and **Support Vector Machine** (radial basis function kernel with tuned cost and gamma).

To address overfitting concerns, we performed 200 repeated stratified 80/20 Monte Carlo cross-validation splits^4,5^. For each split, all five models were fit with class weighting (inverse class frequency) during training; the natural imbalanced distribution was preserved in each test set. Hyperparameters were tuned via 5-fold CV on the initial training set and held fixed across the 200 splits. Reported metrics are mean ± SD of ROC AUC and PR AUC across the 200 test sets (Table S10).

These analyses were intended as pragmatic benchmarking rather than formal clinical prediction model development. Given the class imbalance (~85% non-attempters), we report Precision-Recall AUC (PR AUC) in addition to ROC AUC, as PR AUC is more informative for imbalanced outcomes^6^, and we did not report accuracy, which is misleading with class imbalance.

***Kaplan–Meier Survival Curves***

Survival curves were constructed for the overall sample and stratified by significant risk factors from multivariable Cox regression. For continuous risk factors, stratification cutpoints were selected to maximise interpretability: threat exposure used +1 SD above the mean as a single cutpoint (low/average vs high). Statistical comparisons used log-rank tests appropriate for the counting-process data structure with delayed entry.

***Threat Components by Informant***

To assess whether associations between specific threatening experiences and suicide attempts differed by informant source, we analysed threat-related indicators stratified by reporter: child self-report and parent/caregiver report. Each component was examined using all three analytical approaches (logistic, Cox, quasi-Poisson) with bootstrap multivariable inference. This analysis addresses potential reporter bias and identifies which specific experiences and informant perspectives are most strongly associated with suicide attempt risk (Table S17).

***Lethality Analyses***

Lethality was assessed using the C-SSRS lethality subscale, which rates physical damage from 0 (no damage) to 5 (death). High lethality was defined as moderate to severe physical damage (score ≥2), and low lethality as no or minor damage only (score 0–1). Two complementary lethality analyses were performed. The first compared **high-lethality attempters versus non-attempters** (Table S18): n=91 high-lethality attempters (lethality ≥2) were compared to n=1,751 non-attempters, excluding low-lethality attempters from the sample. This analysis identifies risk factors that distinguish more medically serious attempts from no attempt history. The second was a **within-attempter analysis** (Table S19): n=91 high-lethality versus n=196 low-lethality attempters using single-predictor weighted logistic regression. This analysis identifies predictors of lethality among those who attempt, distinct from predictors of attempt occurrence. Cox regression was not included in the within-attempter analysis because all participants experienced the event (attempt); quasi-Poisson was not included because the outcome is binary.

Both analyses used the same weighted methodology as the main analysis (CBPS weights, 1,000 bootstrap iterations, all risk factors included simultaneously where applicable).

***Ideation-Restricted Sensitivity Analysis***

To test whether risk factor associations were specific to the transition from ideation to action or shared with ideation risk, we conducted an ideation-restricted sensitivity analysis comparing attempters (n=309) versus ideators-without-attempt (n=451), excluding participants with no lifetime suicidal ideation (Table S20). Single-predictor and bootstrap multivariable logistic regression used the same predictor set as the main analysis. Cox regression was not included because there is no differential timing in this cross-sectional comparison; quasi-Poisson was not included because the outcome is binary. Predictors significant in both the main and the ideation-restricted analyses can be interpreted as factors associated with the transition from ideation to action; those significant only in the main analysis are more likely shared with ideation risk. Importantly, ideation was ascertained concurrently with the outcome at Wave 3 via lifetime recall; temporal ordering between ideation onset and attempt cannot be established, and results should be interpreted as cross-sectional differentiation rather than prospective prediction of the ideation-to-action transition.

***Population Attributable Fractions***

Population Attributable Fractions (PAF) were calculated using Levin’s formula:

*PAF = Pₘ(OR−1) / [1 + Pₘ(OR−1)]*

where Pₘ is the prevalence of the exposure in the analytic sample (population prevalence) and OR is the minimally adjusted odds ratio (adjusted for sex and age only) from logistic regression. Minimally adjusted estimates better approximate the total population impact, including both direct and mediated effects; PAFs using fully adjusted ORs are presented as a sensitivity analysis (Table S9, Panel A). PAF represents the proportion of cases theoretically preventable if the exposure were eliminated, assuming a causal relationship.

PAFs were calculated for: (a) main modifiable risk factors (Table 3); (b) individual threat components by informant (Table S14). Bootstrap resampling (1,000 iterations) provided 95% confidence intervals. For continuous variables (childhood threat), exposure was defined as >1 SD above the mean; sensitivity to alternative thresholds (>0·5 SD, >1·0 SD, >1·5 SD) is reported in Table S9, Panel B.

Because the cumulative incidence of suicide attempts in this cohort (15·0%) is not rare, OR-based Levin PAFs may slightly overestimate risk-ratio-based attributable fractions; PAFs are therefore interpreted as approximate population-impact metrics under assumptions of causality, no residual confounding, and no interaction among exposures, rather than directly preventable proportions of cases. PAFs are not defined for protective factors (OR<1) and are reported as N/A in Table 3 for caregiver externalising disorder.

***Directed Acyclic Graph (DAG)***

A DAG presenting the hypothesised causal structure across temporal layers (genetic → perinatal → sociodemographic/family → adversity/cognition → clinical → outcome) is provided as Figure S1. The DAG clarifies which variables function as confounders versus potential mediators in the multivariable models. Because all predictors are entered simultaneously, multivariable coefficients represent associations after mutual adjustment (analogous to direct effects in a causal framework, although observational associations do not establish causality); single-predictor estimates capture total associations including mediated pathways.

**References**

1. Imai K, Ratkovic M. Covariate balancing propensity score. J R Stat Soc Series B. 2014;76(1):243–263.

2. Efron B, Tibshirani RJ. An Introduction to the Bootstrap. Chapman & Hall/CRC; 1994.

3. Boos DD, Stefanski LA. Essential Statistical Inference: Theory and Methods. Springer; 2013.

4. Harrell FE Jr. Regression Modeling Strategies. 2nd ed. Springer; 2015.

5. Steyerberg EW, Harrell FE Jr. Prediction models need appropriate internal, internal–external, and external validation. J Clin Epidemiol. 2016;69:245–247.

6. Saito T, Rehmsmeier M. The precision-recall plot is more informative than the ROC plot when evaluating binary classifiers on imbalanced datasets. PLoS One. 2015;10(3):e0118432.

# Table S1. Bootstrap Diagnostics

**Panel A. Bootstrap Convergence**

| **Model** | **N total** | **N converged** | **N failed** | **Failure rate (%)** |
| --- | --- | --- | --- | --- |
| Logistic regression (LR) | 1,000 | 1,000 | 0 | 0 |
| Cox proportional hazards | 1,000 | 1,000 | 0 | 0 |
| Quasi-Poisson (QP) | 1,000 | 1,000 | 0 | 0 |

*B = 1,000 iterations. Mean of log-scale bootstrap (exponentiated). 95% CI: 2.5th–97.5th percentiles of log-scale (exponentiated).*

**Panel B. Monte Carlo Standard Error of CI Endpoints and Bootstrap Distribution Skewness (Logistic Regression)**

| **Predictor** | **Log skewness** | **MCSE CI low** | **MCSE CI high** |
| --- | --- | --- | --- |
| Maternal alcohol use in pregnancy | -0.066 | 0.0008 | 0.0008 |
| Any externalising disorder | -0.132 | 0.0009 | 0.0009 |
| Any internalising disorder | 0.076 | 0.0010 | 0.0010 |
| Breastfeeding | 0.293 | 0.0013 | 0.0013 |
| Caregiver externalising disorder | -2.108 | 0.0044 | 0.0044 |
| Caregiver internalising disorder | -0.155 | 0.0008 | 0.0008 |
| Caregiver suicide attempt | 0.095 | 0.0010 | 0.0010 |
| Caregiver thought disorder | -0.058 | 0.0012 | 0.0012 |
| Childhood deprivation (z) | 0.054 | 0.0004 | 0.0004 |
| Executive function (z) | 0.277 | 0.0004 | 0.0004 |
| IQ (z) | -0.242 | 0.0004 | 0.0004 |
| Low birthweight (<2500g) | -0.247 | 0.0012 | 0.0012 |
| Maternal smoking in pregnancy | -0.105 | 0.0009 | 0.0009 |
| Neonatal ICU admission | -0.204 | 0.0012 | 0.0012 |
| Eclampsia | -0.121 | 0.0009 | 0.0009 |
| Gestational diabetes | -0.311 | 0.0014 | 0.0014 |
| Other pregnancy complication | -0.322 | 0.0014 | 0.0014 |
| UTI in pregnancy | 0.084 | 0.0009 | 0.0009 |
| Prematurity | 0.097 | 0.0011 | 0.0011 |
| Prenatal care ≥8 visits | 0.061 | 0.0008 | 0.0008 |
| PRS Anxiety (z) | 0.074 | 0.0007 | 0.0007 |
| PRS Depression (z) | 0.045 | 0.0006 | 0.0006 |
| PRS Suicide Attempt (z) | 0.052 | 0.0008 | 0.0008 |
| PRS Suicide Death (z) | 0.063 | 0.0007 | 0.0007 |
| PRS Wellbeing (z) | -0.071 | 0.0005 | 0.0005 |
| Race (white vs non-white) | 0.041 | 0.0009 | 0.0009 |
| Recent DSH/suicidal talk | -0.198 | 0.0014 | 0.0014 |
| Sex (female) | -0.082 | 0.0006 | 0.0006 |
| Childhood threat (z) | -0.045 | 0.0004 | 0.0004 |

*MCSE = Monte Carlo standard error. Most predictors showed approximately symmetric bootstrap distributions on the log scale, supporting the use of the bootstrap mean as the point estimate; caregiver externalising disorder (log skewness −2·108) was an exception, reflecting its very low prevalence (1·3% in the analytic sample). Monte Carlo standard errors of CI endpoints remained small (<0·005) for all predictors, indicating stable CI estimation.*

# Table S2. Variance Inflation Factors (GVIF) for Multicollinearity Assessment

| **Variable** | **GVIF** | **GVIF^(1/2Df)** |
| --- | --- | --- |
| PRS Depression (z) | 4.36 | 2.09 |
| PRS Anxiety (z) | 3.18 | 1.78 |
| PRS Wellbeing (z) | 1.61 | 1.27 |
| PRS Suicide Attempt (z) | 4.29 | 2.07 |
| PRS Suicide Death (z) | 2.03 | 1.42 |
| Sex (female) | 1.21 | 1.10 |
| Race (white vs non-white) | 1.15 | 1.07 |
| Childhood threat (z) | 1.42 | 1.19 |
| Childhood deprivation (z) | 1.32 | 1.15 |
| IQ (z) | 1.30 | 1.14 |
| Executive function (z) | 1.13 | 1.06 |
| Caregiver internalising disorder | 1.21 | 1.10 |
| Caregiver externalising disorder | 1.04 | 1.02 |
| Caregiver suicide attempt | 1.18 | 1.09 |
| Caregiver thought disorder | 1.16 | 1.08 |
| Any internalising disorder | 1.18 | 1.09 |
| Any externalising disorder | 1.16 | 1.08 |
| Recent DSH/suicidal talk | 1.10 | 1.05 |
| Maternal smoking in pregnancy | 1.18 | 1.09 |
| Maternal alcohol use in pregnancy | 1.14 | 1.07 |
| Low birthweight (<2500g) | 1.41 | 1.19 |
| Prematurity | 1.50 | 1.22 |
| Breastfeeding | 1.04 | 1.02 |
| Prenatal care ≥8 visits | 1.08 | 1.04 |
| Neonatal ICU admission | 1.10 | 1.05 |
| Eclampsia | 1.15 | 1.07 |
| Gestational diabetes | 1.07 | 1.03 |
| UTI in pregnancy | 1.10 | 1.05 |
| Other pregnancy complication | 1.07 | 1.03 |

*GVIF = generalised variance inflation factor (car::vif in R). For categorical variables with df>1, GVIF^(1/2Df) is reported. Values >5 indicate moderate, >10 high multicollinearity. All values <5; only PRSs were >2.*

# Table S3. Cox Sensitivity Analysis: Restricted to Attempters with Confirmed C-SSRS Dates

| **Variable** | **Full Sample HR (95% CI)** | **Confirmed Dates HR (95% CI)** |
| --- | --- | --- |
| Sex (female) | 2.88 (2.25–3.69) | 2.90 (2.19–3.96) |
| Race (white vs non-white) | 1.14 (0.86–1.51) | 1.15 (0.82–1.62) |
| Childhood threat (z-score) | 1.21 (1.06–1.38) | 1.23 (1.05–1.45) |
| Childhood deprivation (z-score) | 0.95 (0.83–1.09) | 0.99 (0.84–1.14) |
| IQ (z-score) | 1.06 (0.92–1.23) | 0.99 (0.84–1.16) |
| Executive function (z-score) | 0.93 (0.79–1.09) | 0.98 (0.83–1.17) |
| Caregiver internalising disorder | 1.03 (0.78–1.38) | 1.00 (0.72–1.36) |
| Caregiver externalising disorder | 0.03 (0.00–0.60) | 0.03 (0.00–0.85) |
| Caregiver suicide attempt | 1.88 (1.34–2.63) | 1.87 (1.26–2.66) |
| Caregiver thought disorder | 1.01 (0.67–1.57) | 1.00 (0.63–1.56) |
| Any internalising disorder | 0.96 (0.66–1.36) | 1.01 (0.64–1.48) |
| Any externalising disorder | 1.50 (1.07–2.08) | 1.47 (0.97–2.15) |
| Recent DSH/suicidal talk | 1.17 (0.64–2.03) | 0.97 (0.43–1.88) |
| Maternal smoking in pregnancy | 1.18 (0.84–1.58) | 1.25 (0.89–1.72) |
| Maternal alcohol use in pregnancy | 1.35 (1.01–1.77) | 1.51 (1.07–2.07) |
| Low birthweight (<2500g) | 1.04 (0.65–1.62) | 1.07 (0.60–1.83) |
| Prematurity | 1.09 (0.76–1.58) | 0.90 (0.55–1.40) |
| Breastfeeding | 1.26 (0.78–2.22) | 1.13 (0.70–2.02) |
| Prenatal care ≥8 visits | 1.06 (0.78–1.49) | 1.14 (0.85–1.58) |
| Neonatal ICU admission | 0.68 (0.41–1.07) | 0.89 (0.53–1.49) |
| Eclampsia | 0.90 (0.63–1.23) | 0.83 (0.56–1.18) |
| Gestational diabetes | 1.76 (0.95–2.86) | 2.12 (1.14–3.53) |
| UTI in pregnancy | 0.96 (0.67–1.29) | 0.86 (0.57–1.23) |
| Other pregnancy complication | 0.80 (0.43–1.33) | 0.83 (0.43–1.39) |
| PRS Anxiety (z-score) | 0.97 (0.77–1.23) | 1.02 (0.79–1.32) |
| PRS Depression (z-score) | 1.24 (0.96–1.61) | 1.27 (0.93–1.72) |
| PRS Suicide Attempt (z-score) | 0.88 (0.65–1.21) | 0.88 (0.62–1.24) |
| PRS Suicide Death (z-score) | 0.90 (0.74–1.10) | 0.88 (0.69–1.09) |
| PRS Wellbeing (z-score) | 0.91 (0.79–1.04) | 0.90 (0.77–1.05) |

*Full sample: N = 2,049 (all attempters and non-attempters in the Cox analytic sample). Confirmed dates: N = 1,980, excluding 69 attempters with DAWBA-imputed timing.*

*Bootstrap with 1,000 iterations on the log-hazard scale. Estimates were substantively unchanged across all predictors, with the exception that maternal alcohol use in pregnancy and gestational diabetes strengthened modestly when restricted to confirmed dates.*

# Table S4. CBPS Diagnostics and Trimming Sensitivity

**Panel A. Weight Distribution**

| **Statistic** | **Value** |
| --- | --- |
| N | 2,060 |
| Mean | 2.0571 |
| Median | 1.5743 |
| SD | 1.8089 |
| Min | 1.0153 |
| Max | 32.0470 |
| IQR | 0.7266 |
| 1st percentile | 1.0599 |
| 5th percentile | 1.1300 |
| 95th percentile | 4.4566 |
| 99th percentile | 10.2439 |

**Panel B. Sensitivity to Weight Trimming**

| **Weights** | **Mean** | **SD** | **Min** | **Max** | **ESS** |
| --- | --- | --- | --- | --- | --- |
| Original CBPS | 2.0571 | 1.8089 | 1.0153 | 32.0470 | 1,162.0 |
| Trimmed 1/99 | 2.0034 | 1.3550 | 1.0599 | 10.2439 | 1,413.6 |
| Trimmed 5/95 | 1.8864 | 0.8584 | 1.1300 | 4.4566 | 1,706.8 |

**Panel C. Standardised Mean Differences (High-risk vs Random Subsamples)**

| **Variable** | **SMD Unweighted** | **SMD Weighted** |
| --- | --- | --- |
| Age (z) | 0.205 | 0.084 |
| Sex (female) | 0.055 | 0.020 |
| Race (white vs non-white) | 0.005 | 0.046 |
| Childhood threat (z) | 0.346 | 0.125 |
| Childhood deprivation (z) | 0.197 | 0.135 |
| IQ (z) | 0.206 | 0.115 |
| Executive function (z) | 0.069 | 0.064 |

*CBPS = covariate-balancing propensity score. ESS = effective sample size. SMDs <0·10 indicate adequate balance; weighting reduced imbalance across variables, although modest residual imbalance remained for childhood threat (SMD 0·125), childhood deprivation (0·135), and IQ (0·115).*

# Table S5. Per-Variable Missingness in the Analytic Sample (N=2,060)

| **Variable** | **N total** | **N valid** | **N missing** | **% missing** |
| --- | --- | --- | --- | --- |
| Executive function (z) | 2,060 | 1,622 | 438 | 21.3 |
| Prenatal care ≥8 visits | 2,060 | 1,734 | 326 | 15.8 |
| PRS Depression (z) | 2,060 | 1,817 | 243 | 11.8 |
| PRS Anxiety (z) | 2,060 | 1,817 | 243 | 11.8 |
| PRS Wellbeing (z) | 2,060 | 1,817 | 243 | 11.8 |
| PRS Suicide Attempt (z) | 2,060 | 1,817 | 243 | 11.8 |
| PRS Suicide Death (z) | 2,060 | 1,817 | 243 | 11.8 |
| IQ (z) | 2,060 | 1,864 | 196 | 9.5 |
| Low birthweight (<2500g) | 2,060 | 1,950 | 110 | 5.3 |
| Prematurity | 2,060 | 2,022 | 38 | 1.8 |
| UTI in pregnancy | 2,060 | 2,022 | 38 | 1.8 |
| Eclampsia | 2,060 | 2,034 | 26 | 1.3 |
| Gestational diabetes | 2,060 | 2,042 | 18 | 0.9 |
| Other pregnancy complication | 2,060 | 2,048 | 12 | 0.6 |
| Maternal alcohol use in pregnancy | 2,060 | 2,052 | 8 | 0.4 |
| Recent DSH/suicidal talk | 2,060 | 2,056 | 4 | 0.2 |
| Maternal smoking in pregnancy | 2,060 | 2,058 | 2 | 0.1 |
| Neonatal ICU admission | 2,060 | 2,058 | 2 | 0.1 |
| Race (white vs non-white) | 2,060 | 2,059 | 1 | 0.0 |
| Sex (female) | 2,060 | 2,060 | 0 | 0.0 |
| Caregiver internalising disorder | 2,060 | 2,060 | 0 | 0.0 |
| Caregiver externalising disorder | 2,060 | 2,060 | 0 | 0.0 |
| Caregiver suicide attempt | 2,060 | 2,060 | 0 | 0.0 |
| Caregiver thought disorder | 2,060 | 2,060 | 0 | 0.0 |
| Childhood threat (z) | 2,060 | 2,060 | 0 | 0.0 |
| Childhood deprivation (z) | 2,060 | 2,060 | 0 | 0.0 |
| Any internalising disorder | 2,060 | 2,060 | 0 | 0.0 |
| Any externalising disorder | 2,060 | 2,060 | 0 | 0.0 |
| Breastfeeding | 2,060 | 2,060 | 0 | 0.0 |

*MICE: m=5 imputations, 10 iterations. Predictive mean matching (continuous), logistic regression (binary). Outcome variables were not imputed.*

# Table S6. IPAW Sensitivity Analysis (CBPS vs CBPS × IPAW Combined Weights)

| **Variable** | **OR (CBPS)** | **p (CBPS)** | **OR (Combined)** | **p (Combined)** | **OR change (%)** |
| --- | --- | --- | --- | --- | --- |
| PRS Depression (z) | 1.450 | 0.014 | 1.471 | 0.011 | 1.5 |
| PRS Anxiety (z) | 1.218 | 0.149 | 1.235 | 0.124 | 1.4 |
| PRS Wellbeing (z) | 0.887 | 0.101 | 0.889 | 0.111 | 0.3 |
| PRS Suicide Attempt (z) | 0.767 | 0.093 | 0.751 | 0.070 | -2.1 |
| PRS Suicide Death (z) | 0.769 | 0.021 | 0.771 | 0.022 | 0.2 |
| Maternal smoking in pregnancy | 1.409 | 0.052 | 1.397 | 0.060 | -0.9 |
| Maternal alcohol use in pregnancy | 1.314 | 0.093 | 1.296 | 0.114 | -1.4 |
| Low birthweight (<2500g) | 1.507 | 0.072 | 1.520 | 0.068 | 0.9 |
| Prematurity | 0.840 | 0.422 | 0.825 | 0.380 | -1.8 |
| Breastfeeding | 1.200 | 0.490 | 1.240 | 0.423 | 3.3 |
| Prenatal care ≥8 visits | 1.279 | 0.115 | 1.261 | 0.138 | -1.4 |
| Neonatal ICU admission | 0.851 | 0.502 | 0.839 | 0.470 | -1.4 |
| Eclampsia | 0.700 | 0.059 | 0.715 | 0.077 | 2.1 |
| Gestational diabetes | 2.382 | 0.004 | 2.360 | 0.005 | -0.9 |
| UTI in pregnancy | 1.106 | 0.550 | 1.134 | 0.453 | 2.6 |
| Other pregnancy complication | 0.862 | 0.617 | 0.844 | 0.572 | -2.1 |
| Sex (female) | 3.265 | <0.001 | 3.234 | <0.001 | -1.0 |
| Race (white vs non-white) | 1.296 | 0.129 | 1.294 | 0.132 | -0.1 |
| Caregiver internalising disorder | 0.988 | 0.942 | 0.977 | 0.890 | -1.1 |
| Caregiver externalising disorder | 0.239 | 0.085 | 0.245 | 0.083 | 2.5 |
| Caregiver suicide attempt | 1.980 | <0.001 | 1.982 | <0.001 | 0.1 |
| Caregiver thought disorder | 0.873 | 0.554 | 0.876 | 0.568 | 0.3 |
| Childhood threat (z) | 1.361 | <0.001 | 1.369 | <0.001 | 0.6 |
| Childhood deprivation (z) | 0.873 | 0.088 | 0.873 | 0.090 | 0.0 |
| IQ (z) | 1.017 | 0.832 | 1.019 | 0.807 | 0.3 |
| Executive function (z) | 1.039 | 0.620 | 1.035 | 0.657 | -0.4 |
| Any internalising disorder | 0.901 | 0.626 | 0.909 | 0.661 | 0.9 |
| Any externalising disorder | 1.487 | 0.035 | 1.430 | 0.058 | -3.8 |
| Recent DSH/suicidal talk | 1.263 | 0.466 | 1.264 | 0.470 | 0.1 |

*CBPS = selection weights only. Combined = CBPS × IPAW (inverse probability of attrition weights). Trimmed at 1st/99th percentiles. All OR changes were <4%, indicating that differential attrition did not materially bias findings.*

# Table S7. PRS Composite (PC1) and Suicide Attempts

| **Outcome** | **Estimate (95% CI)** | **Comparison** |
| --- | --- | --- |
| Cumulative incidence (LR, OR) | 1.28 (1.08–1.53) | Significant |
| Onset timing (Cox, HR) | 1.26 (1.01–1.56) | Significant |
| Number of attempts (QP, IRR) | 1.01 (0.84–1.23) | Not significant |

*PC1 = first principal component of the five PRS, capturing 63·6% of variance. Loadings: depression 0·52, anxiety 0·50, suicide attempt 0·51, suicide death 0·38, well-being −0·27. Models adjusted for all other risk factors. PRS-derived PC1 substituted for the five individual PRS.*

# Table S8. Sequential Model-Building with Incremental Variance by Domain

| **Step** | **Domain** | **N variables added** | **N variables cumulative** | **Pseudo-R²** | **ΔR²** |
| --- | --- | --- | --- | --- | --- |
| 1 | Sociodemographic | 2 | 2 | 0.0926 | 0.0926 |
| 2 | Adversity | 2 | 4 | 0.1367 | 0.0441 |
| 3 | Family | 4 | 8 | 0.1542 | 0.0175 |
| 4 | Clinical | 3 | 11 | 0.1593 | 0.0051 |
| 5 | Perinatal | 11 | 22 | 0.1844 | 0.0251 |
| 6 | Cognitive | 2 | 24 | 0.1910 | 0.0066 |
| 7 | Genetic | 15 | 39 | 0.2427 | 0.0517 |

*Weighted logistic regression (quasibinomial). Domains added cumulatively in temporal order. Pseudo-R² = Nagelkerke. ΔR² = change from previous step. Likelihood ratio tests are not valid under survey weighting; differences are descriptive.*

# Table S9. Sensitivity Analyses for Population Attributable Fractions

**Panel A. Comparison of Minimally vs Fully Adjusted ORs**

| **Variable** | **OR minimal** | **PAF minimal (%)** | **OR full** | **PAF full (%)** | **Difference (%)** |
| --- | --- | --- | --- | --- | --- |
| Any internalising disorder | 1.37 | 4.5 | 0.90 | N/A | — |
| Any externalising disorder | 1.82 | 10.2 | 1.49 | 6.3 | 3.9 |
| Recent DSH/suicidal talk | 1.80 | 2.9 | 1.26 | 1.0 | 1.9 |
| Caregiver internalising disorder | 1.45 | 11.6 | 0.99 | N/A | — |
| Caregiver externalising disorder | 0.43 | N/A | 0.24 | N/A | — |
| Caregiver suicide attempt | 2.35 | 14.1 | 1.98 | 10.7 | 3.4 |
| Childhood threat (z-score) | 1.79 | 11.5 | 1.36 | 5.6 | 5.9 |

*Minimal: ORs adjusted for sex and age only. Full: ORs adjusted for all predictors entered simultaneously in the multivariable model. Fully adjusted ORs may underestimate population impact if the adjustment set includes mediators on the causal pathway. PAF is not defined when OR ≤ 1; rows with fully adjusted OR < 1 (any internalising, caregiver internalising, caregiver externalising) are reported as N/A in the corresponding column.*

**Panel B. Sensitivity to Childhood Threat Threshold**

| **Threshold** | **N exposed** | **Prevalence (population, %)** | **Prevalence (cases, %)** | **OR** | **PAF (%)** |
| --- | --- | --- | --- | --- | --- |
| >0.5 SD | 571 | 27.7 | 35.3 | 1.55 | 13.3 |
| >1.0 SD | 339 | 16.5 | 23.3 | 1.79 | 11.5 |
| >1.5 SD | 187 | 9.1 | 14.9 | 2.13 | 9.3 |

*PAFs computed using Levin’s formula with population prevalence. Estimates were stable across thresholds; the >1 SD threshold was used as the primary specification (Table 3).*

# Table S10. Machine Learning Model Performance Across 200 Repeated Stratified Splits

| **Model** | **ROC AUC (mean ± SD)** | **PR AUC (mean ± SD)** | **N splits** |
| --- | --- | --- | --- |
| Logistic regression | 0.665 ± 0.033 | 0.261 ± 0.046 | 200 |
| Elastic Net | 0.652 ± 0.040 | 0.234 ± 0.047 | 200 |
| Random Forest | 0.626 ± 0.033 | 0.220 ± 0.038 | 200 |
| XGBoost | 0.598 ± 0.037 | 0.207 ± 0.034 | 200 |
| Support Vector Machine | 0.606 ± 0.034 | 0.220 ± 0.038 | 200 |

*200 repeated stratified 80/20 splits with class weighting (inverse class frequency). Hyperparameters tuned via 5-fold CV on the initial training set and held fixed across splits. AUC = area under the curve; PR = precision–recall; SD = standard deviation.*

# Table S11. Participant Flow

| **Stage** | **N** | **Reason / Notes** |
| --- | --- | --- |
| Initial screening (Family History Screen) | 9,937 | — |
| Selected for deep phenotyping (Wave 0) | 2,511 | 958 random + 1,553 high-risk |
| Pre-baseline suicide attempts excluded | 16 | Excluded (incident outcome) |
| Eligible cohort with complete baseline | 2,495 | — |
| Lost to follow-up by Wave 3 | 435 | 17.4% attrition |
| **Wave 3 completers (analytic sample)** | **2,060** | Logistic and quasi-Poisson analyses |
| Excluded from Cox (no survival timing) | 11 | Missing date and DAWBA timing |
| **Cox analytic sample** | **2,049** | 298 incident events |

**Table S12. Comparison of Total Enrolled Cohort vs Analytic Sample**

| **Variable** | **Total cohort (N=2,511)** | **Analytic sample (N=2,060)** | **p-value** |
| --- | --- | --- | --- |
| **GENETIC** |  |  |  |
| PRS Depression (z) | 0 (1) | 0.01 (0.99) | 0.82 |
| PRS Anxiety (z) | 0 (1) | 0.01 (1) | 0.85 |
| PRS Wellbeing (z) | 0 (1) | 0 (1.01) | 0.89 |
| PRS Suicide Attempts (z) | 0 (1) | 0 (0.99) | 0.91 |
| PRS Suicide Death (z) | 0 (1) | 0.02 (1.01) | 0.59 |
| **PERINATAL** |  |  |  |
| Maternal smoking in pregnancy | 420 (16.7%) | 334 (16.2%) | 0.66 |
| Maternal alcohol use in pregnancy | 545 (21.8%) | 451 (21.9%) | 0.96 |
| Low birthweight (<2500g) | 243 (10.3%) | 219 (10.6%) | 0.72 |
| Prematurity | 365 (14.8%) | 309 (15.0%) | 0.89 |
| Breastfeeding | 2,296 (91.4%) | 1,890 (91.7%) | 0.75 |
| Prenatal care ≥8 visits | 1,494 (70.7%) | 1,454 (70.7%) | 1.00 |
| Neonatal ICU admission | 292 (11.6%) | 234 (11.4%) | 0.80 |
| Eclampsia | 484 (19.5%) | 404 (19.6%) | 0.97 |
| Gestational diabetes | 107 (4.3%) | 90 (4.4%) | 0.98 |
| UTI in pregnancy | 472 (19.2%) | 396 (19.2%) | 0.98 |
| Other pregnancy complication | 136 (5.5%) | 113 (5.5%) | 1.00 |
| **SOCIODEMOGRAPHIC** |  |  |  |
| Female sex | 1,135 (45.2%) | 989 (48.0%) | 0.06 |
| Non-white race | 1,103 (43.9%) | 904 (43.9%) | 0.99 |
| **FAMILY (MAIN CAREGIVER)** |  |  |  |
| Caregiver internalising disorder | 719 (28.6%) | 598 (29.0%) | 0.79 |
| Caregiver externalising disorder | 35 (1.4%) | 26 (1.3%) | 0.80 |
| Caregiver suicide attempt | 292 (11.6%) | 251 (12.2%) | 0.60 |
| Caregiver thought disorder | 266 (10.6%) | 216 (10.5%) | 0.94 |
| **ADVERSITY** |  |  |  |
| Threat exposure (z) | 0 (1) | 0 (1) | 0.95 |
| Deprivation (z) | 0 (1) | −0.03 (1) | 0.36 |
| **COGNITIVE** |  |  |  |
| IQ (z) | 0 (1) | 0.03 (1) | 0.35 |
| Executive function (z) | 0 (1) | 0.01 (0.99) | 0.72 |
| **CLINICAL (Wave 0)** |  |  |  |
| Any internalising disorder | 302 (12.0%) | 261 (12.7%) | 0.54 |
| Any externalising disorder | 353 (14.1%) | 285 (13.8%) | 0.86 |

*Values are n (%) for categorical variables and mean (SD) for continuous variables. Total enrolled cohort: N=2,511; analytic sample: N=2,060. P-values are from proportion tests for categorical variables and t-tests for continuous variables. Percentages are unweighted.*

# Table S13. Unweighted Multivariable Bootstrap Models (Selected Predictors)

| **Variable** | **LR OR (95% CI)** | **Cox HR (95% CI)** | **QP IRR (95% CI)** |
| --- | --- | --- | --- |
| Sex (female) | 3.05 (2.27–4.10) | 2.92 (2.30–3.71) | 3.10 (2.21–4.43) |
| Caregiver suicide attempt | 2.06 (1.41–3.05) | 1.86 (1.34–2.59) | 1.30 (0.84–2.00) |
| Childhood threat (z) | 1.27 (1.10–1.46) | 1.22 (1.07–1.39) | 1.27 (1.06–1.51) |
| Any externalising disorder | 1.51 (1.05–2.13) | 1.51 (1.07–2.07) | 1.36 (0.90–1.96) |
| Maternal alcohol use in pregnancy | 1.36 (0.99–1.89) | 1.34 (1.00–1.76) | 1.13 (0.82–1.55) |
| Gestational diabetes | 1.89 (1.01–3.25) | 1.74 (0.96–2.83) | 2.16 (1.13–3.80) |
| PRS Depression (z) | 1.27 (0.94–1.69) | 1.27 (0.99–1.62) | 1.40 (1.03–1.92) |
| PRS Anxiety (z) | 1.07 (0.83–1.36) | 1.00 (0.82–1.27) | 0.79 (0.59–1.06) |
| PRS Wellbeing (z) | 0.91 (0.79–1.05) | 0.94 (0.83–1.06) | 1.04 (0.90–1.21) |
| PRS Suicide Attempt (z) | 0.89 (0.65–1.21) | 0.89 (0.66–1.22) | 1.20 (0.79–1.78) |
| PRS Suicide Death (z) | 0.89 (0.71–1.12) | 0.91 (0.74–1.11) | 0.74 (0.58–0.93) |
| Childhood deprivation (z) | 0.96 (0.82–1.13) | 0.95 (0.83–1.09) | 0.90 (0.76–1.07) |
| Caregiver internalising disorder | 1.04 (0.74–1.41) | 1.04 (0.79–1.39) | 0.96 (0.66–1.39) |
| Caregiver thought disorder | 0.99 (0.60–1.55) | 1.00 (0.66–1.55) | 1.07 (0.65–1.78) |
| Any internalising disorder | 0.93 (0.65–1.39) | 0.97 (0.67–1.38) | 0.84 (0.52–1.27) |
| Recent DSH/suicidal talk | 1.21 (0.62–2.18) | 1.16 (0.64–2.02) | 1.39 (0.61–2.51) |
| IQ (z) | 1.05 (0.88–1.22) | 1.06 (0.92–1.23) | 0.86 (0.69–1.08) |
| Executive function (z) | 0.99 (0.84–1.20) | 0.93 (0.79–1.09) | 1.11 (0.86–1.45) |
| Maternal smoking in pregnancy | 1.16 (0.79–1.65) | 1.17 (0.83–1.56) | 1.10 (0.75–1.61) |
| Race (white vs non-white) | 1.16 (0.83–1.63) | 1.13 (0.85–1.51) | 1.21 (0.78–1.84) |

*Unweighted analyses (without CBPS or IPAW) are presented as a robustness check. Estimates were concordant with the main weighted analyses.*

# Table S14. Population Attributable Fractions for Threat Components by Informant

**Panel A. Child-Reported Exposures**

| **Threat component** | **N** | **Population Prevalence** | **Prevalence in Cases** | **OR (95% CI)** | **PAF (%)** | **Sig.** |
| --- | --- | --- | --- | --- | --- | --- |
| Bullying (child report) | 2,058 | 32.9% | 44.6% | 1.95 (1.64–2.33) | 23.8% | *** |
| Emotional abuse (child report) | 2,058 | 22.9% | 27.0% | 1.41 (1.16–1.72) | 8.6% | *** |
| Physical abuse (child report) | 2,058 | 15.1% | 17.9% | 1.50 (1.19–1.89) | 7.0% | *** |
| Attack or threat | 2,060 | 4.1% | 5.2% | 2.08 (1.49–2.92) | 4.2% | *** |
| Sexual abuse | 2,059 | 2.5% | 4.9% | 1.74 (1.15–2.64) | 1.8% | ** |

**Panel B. Parent Report**

| **Threat component** | **N** | **Population Prevalence** | **Prevalence in Cases** | **OR (95% CI)** | **PAF (%)** | **Sig.** |
| --- | --- | --- | --- | --- | --- | --- |
| Emotional abuse (parent report) | 2,059 | 44.2% | 51.9% | 1.63 (1.37–1.94) | 21.8% | *** |
| Physical abuse (parent report) | 2,060 | 14.2% | 19.4% | 2.48 (2.01–3.07) | 17.4% | *** |
| Bullying (parent report) | 2,060 | 38.9% | 46.9% | 1.44 (1.21–1.71) | 14.6% | *** |
| Witnessed domestic violence | 2,060 | 7.1% | 9.7% | 1.76 (1.31–2.36) | 5.1% | *** |
| Physical abuse (DAWBA) | 2,060 | 3.4% | 5.8% | 1.73 (1.18–2.54) | 2.4% | ** |
| Witnessed attack | 2,060 | 4.2% | 5.5% | 1.39 (0.96–2.01) | 1.6% |  |

*PAF = Population Attributable Fraction calculated using Levin’s formula: PAF = P_pop(OR−1) / [1 + P_pop(OR−1)], where P_pop is the population prevalence of the exposure in the analytic sample (column 3). Prevalence in cases (column 4) is provided for descriptive comparison and is not used in the formula. OR adjusted for sex and age and weighted by CBPS weights.*

*Panel A: threat exposures reported by the child. Panel B: threat exposures reported by parent/caregiver or DAWBA. Population prevalences are pooled across the five multiple imputation datasets and are derived from the same imputed analytic sample used for the bootstrap PAF estimates.*

** p < 0.05, ** p < 0.01, *** p < 0.001.*

# Table S15. Baseline Characteristics of Wave 3 Completers (N=2,060) vs Non-Completers (N=451)

| **Variable** | **Completers** | **Non-completers** | **p-value** |
| --- | --- | --- | --- |
| Female sex (%) | 48.0 | 39.5 | <0.001 |
| Mean baseline age (years, SD) | 10.4 (1.9) | 10.5 (1.9) | 0.51 |
| Non-white race (%) | 43.9 | 41.5 | 0.34 |
| Childhood threat (z, mean SD) | -0.00 (1.00) | 0.05 (1.10) | 0.31 |
| Childhood deprivation (z, mean SD) | -0.03 (1.00) | 0.14 (1.05) | 0.004 |
| IQ (z, mean SD) | 0.03 (1.00) | -0.10 (0.99) | 0.018 |
| Executive function (z, mean SD) | 0.01 (0.99) | -0.03 (1.02) | 0.46 |
| Any internalising disorder (%) | 12.7 | 11.3 | 0.43 |
| Any externalising disorder (%) | 13.8 | 15.7 | 0.31 |
| Caregiver suicide attempt (%) | 12.2 | 12.0 | 0.92 |

*Comparison between Wave 3 completers (N=2,060) and non-completers (N=451) within the original deeply phenotyped cohort (N=2,511; non-completers therefore include the 16 participants with pre-baseline suicide attempts who were excluded from incident-risk analyses). Differential attrition was observed for sex, deprivation, and IQ. IPAW (Table S6) was applied to assess robustness to differential attrition.*

# Table S16. Proportional Hazards Assumption Test (Schoenfeld Residuals)

| **Variable** | **χ²** | **df** | **p-value** |
| --- | --- | --- | --- |
| Sex (female) | 22.84 | 1 | <0.001 |
| Race (white vs non-white) | 1.08 | 1 | 0.299 |
| Childhood threat (z) | 9.42 | 1 | 0.002 |
| Childhood deprivation (z) | 0.35 | 1 | 0.554 |
| IQ (z) | 3.21 | 1 | 0.073 |
| Executive function (z) | 0.84 | 1 | 0.359 |
| Caregiver internalising disorder | 0.16 | 1 | 0.689 |
| Caregiver externalising disorder | 3.01 | 1 | 0.083 |
| Caregiver suicide attempt | 11.78 | 1 | <0.001 |
| Caregiver thought disorder | 0.04 | 1 | 0.842 |
| Any internalising disorder | 5.92 | 1 | 0.015 |
| Any externalising disorder | 7.13 | 1 | 0.008 |
| Recent DSH/suicidal talk | 0.53 | 1 | 0.467 |
| PRS Depression (z) | 2.18 | 1 | 0.140 |
| PRS Anxiety (z) | 1.45 | 1 | 0.229 |
| PRS Wellbeing (z) | 3.62 | 1 | 0.057 |
| PRS Suicide Attempt (z) | 0.21 | 1 | 0.647 |
| PRS Suicide Death (z) | 0.92 | 1 | 0.337 |
| Maternal smoking in pregnancy | 0.28 | 1 | 0.597 |
| Maternal alcohol use in pregnancy | 1.94 | 1 | 0.164 |
| Low birthweight (<2500g) | 0.41 | 1 | 0.522 |
| Prematurity | 0.07 | 1 | 0.791 |
| Breastfeeding | 0.85 | 1 | 0.357 |
| Prenatal care ≥8 visits | 0.62 | 1 | 0.431 |
| Neonatal ICU admission | 3.55 | 1 | 0.060 |
| Eclampsia | 0.49 | 1 | 0.484 |
| Gestational diabetes | 0.10 | 1 | 0.752 |
| UTI in pregnancy | 0.07 | 1 | 0.791 |
| Other pregnancy complication | 1.04 | 1 | 0.308 |
| **GLOBAL** | **171.20** | **39** | **<0.001** |

*Schoenfeld residual test using cox.zph(). Global test indicated non-proportionality (χ²=171.2, df=39, p<0.001). Several predictors showed non-proportionality, including sex, childhood threat, caregiver suicide attempt, and externalising/internalising disorders. The direction of association was consistent for all predictors. Reported HRs are interpreted as weighted average effects across the follow-up period.*

**Table S17. Threat Components by Informant: Single-Predictor and Multivariable Bootstrap Models**

**Panel A. Child-Reported Threat Components**

| **Component** | **Single-pred. OR (95% CI)** | **Single-pred. HR (95% CI)** | **Single-pred. IRR (95% CI)** | **Multivariable OR (95% CI)** | **Multivariable HR (95% CI)** |
| --- | --- | --- | --- | --- | --- |
| Bullying | 1.78 (1.29–2.45)* | 1.78 (1.30–2.44)** | 1.67 (1.02–2.72) | 1.73 (1.22–2.45)* | 1.75 (1.24–2.47)* |
| Emotional abuse | 1.38 (0.98–1.96) | 1.32 (0.94–1.87) | 0.99 (0.62–1.60) | 0.99 (0.68–1.43) | 0.93 (0.63–1.36) |
| Physical abuse | 1.46 (0.98–2.18) | 1.43 (0.97–2.13) | 1.53 (0.83–2.83) | 1.10 (0.73–1.65) | 1.09 (0.72–1.65) |

**Panel B. Parent-Reported and DAWBA-Derived Threat Components**

| **Component** | **Single-pred. OR (95% CI)** | **Single-pred. HR (95% CI)** | **Single-pred. IRR (95% CI)** | **Multivariable OR (95% CI)** | **Multivariable HR (95% CI)** |
| --- | --- | --- | --- | --- | --- |
| Emotional abuse | 1.57 (1.15–2.16)* | 1.49 (1.11–2.01)* | 1.32 (0.79–2.18) | 1.24 (0.90–1.72) | 1.17 (0.84–1.64) |
| Physical abuse (parent questionnaire) | 2.43 (1.58–3.73)** | 2.14 (1.43–3.19)** | 2.09 (1.22–3.60) | 1.96 (1.24–3.10)* | 1.86 (1.18–2.95)* |
| Physical abuse (DAWBA-PTSD) | 1.85 (0.79–4.32) | 1.53 (0.67–3.53) | 1.47 (0.65–3.31) | 0.97 (0.39–2.42) | 0.88 (0.37–2.10) |
| Bullying | 1.41 (1.02–1.95) | 1.38 (1.01–1.87) | 1.45 (0.88–2.38) | 1.09 (0.79–1.51) | 1.08 (0.78–1.50) |
| Witnessed domestic violence | 1.75 (1.03–2.97) | 1.79 (1.13–2.86) | 1.59 (0.71–3.53) | 1.29 (0.76–2.19) | 1.40 (0.86–2.29) |
| Attack or threat | 2.30 (0.95–5.54) | 2.07 (0.96–4.46) | 0.96 (0.50–1.84) | 2.00 (0.79–5.04) | 1.95 (0.74–5.16) |
| Witnessed attack | 1.49 (0.69–3.20) | 1.45 (0.71–2.92) | 1.92 (0.69–5.35) | 0.80 (0.35–1.82) | 0.84 (0.36–1.98) |
| Sexual abuse | 2.08 (0.79–5.48) | 1.92 (0.79–4.71) | 1.20 (0.53–2.72) | 1.46 (0.61–3.49) | 1.56 (0.72–3.41) |

*Multivariable models entered all threat components from both informants simultaneously, with sex and age included as covariates. Child-reported bullying (OR 1.73, HR 1.75) and parent-reported physical abuse (OR 1.96, HR 1.86, IRR 1.87, 95% CI 1.11–3.17) were the components that retained association after mutual adjustment in threat-component models. Effect estimates show point estimate (95% confidence interval). OR = odds ratio (weighted logistic regression); HR = hazard ratio (weighted Cox proportional hazards); IRR = incidence rate ratio (weighted quasi-Poisson). For single-predictor models, * p < 0.05, ** p < 0.01, *** p < 0.001 (FDR-corrected within each model type). Multivariable estimates derived from bootstrap (1,000 iterations); * indicates 95% CI excluding the null (1.00). Variables labelled “(parent questionnaire)” were assessed using BHRC-specific parent-report instruments; variables labelled “(DAWBA-PTSD)” were assessed via the DAWBA PTSD module. Witnessed domestic violence, attack or threat, witnessed attack, and sexual abuse were derived from the DAWBA PTSD module and are reported within Panel B for consistency with their primary informant source.*

**Table S18. High-Lethality Attempters (n=91) vs Non-Attempters (n=1,751): Multivariable Bootstrap Models**

| **Variable** | **LR OR (95% CI)** | **Cox HR (95% CI)** | **QP IRR (95% CI)** |
| --- | --- | --- | --- |
| **Sex (female)** | **3.69 (1.85–7.43)** | **4.80 (2.77–9.18)** | **3.16 (1.74–7.76)** |
| **PRS Depression (z)** | **2.21 (1.09–4.43)** | **1.98 (1.13–3.60)** | **2.68 (1.43–4.97)** |
| PRS Anxiety (z) | 0.95 (0.49–1.84) | 0.83 (0.50–1.40) | 0.65 (0.30–1.34) |
| PRS Wellbeing (z) | 0.86 (0.65–1.13) | 0.85 (0.67–1.07) | 0.84 (0.62–1.13) |
| PRS Suicide Attempt (z) | 0.79 (0.39–1.55) | 0.81 (0.45–1.46) | 1.05 (0.50–2.13) |
| PRS Suicide Death (z) | 0.74 (0.49–1.10) | 0.78 (0.55–1.12) | 0.66 (0.43–0.98) |
| Childhood threat (z) | 1.41 (1.01–1.94) | 1.40 (1.09–1.80) | 1.34 (1.03–1.90) |
| Childhood deprivation (z) | 0.94 (0.68–1.31) | 0.93 (0.71–1.20) | 0.86 (0.59–1.25) |
| Maternal alcohol use in pregnancy | 1.79 (1.00–3.08) | 1.92 (1.12–3.26) | 1.59 (0.83–2.99) |
| Prenatal care ≥8 visits | 2.00 (1.06–4.05) | 1.84 (1.04–3.30) | 1.78 (0.84–3.65) |
| Caregiver suicide attempt | 1.32 (0.62–2.61) | 1.20 (0.63–2.30) | 1.34 (0.62–2.81) |
| Caregiver internalising disorder | 0.98 (0.51–1.81) | 1.00 (0.58–1.69) | 1.04 (0.50–2.17) |
| Any externalising disorder | 1.30 (0.61–2.59) | 1.28 (0.69–2.40) | 1.24 (0.55–2.76) |
| Any internalising disorder | 0.85 (0.41–1.69) | 0.92 (0.51–1.65) | 0.78 (0.36–1.57) |

*Comparison group: 1,751 non-attempters. Bold = robust predictors significant across all three models. Female sex and PRS-depression were the most consistent predictors of medically serious attempts. Childhood threat and maternal alcohol use in pregnancy retained association with onset after mutual adjustment, while caregiver suicide attempts and externalising disorders, associated with overall attempts in multivariable models, were not associated with high-lethality attempts specifically.*

**Table S19. Within-Attempter Analysis: High-Lethality (n=91) vs Low-Lethality (n=196) Attempters**

| **Variable** | **OR (95% CI)** | **p-value** | **Significance** |
| --- | --- | --- | --- |
| PRS Depression (z-score) | 1.61 (1.29–2.05) | <0.001 | *** |
| PRS Anxiety (z-score) | 1.54 (1.24–1.94) | <0.001 | *** |
| UTI in pregnancy | 2.07 (1.40–3.05) | <0.001 | *** |
| PRS Wellbeing (z-score) | 0.72 (0.59–0.87) | 0.001 | ** |
| Caregiver thought disorder | 0.46 (0.27–0.77) | 0.003 | ** |
| Any externalising disorder | 0.54 (0.34–0.84) | 0.005 | ** |
| Caregiver suicide attempt | 0.56 (0.36–0.86) | 0.010 | * |
| PRS Suicide Attempt (z-score) | 1.41 (1.07–1.89) | 0.020 | * |
| Eclampsia | 1.59 (1.02–2.46) | 0.040 | * |
| Prenatal care ≥8 visits | 1.47 (0.97–2.25) | 0.070 |  |
| PRS Suicide Death (z-score) | 0.80 (0.61–1.05) | 0.110 |  |
| Prematurity | 1.38 (0.84–2.23) | 0.200 |  |
| Childhood threat (z-score) | 0.91 (0.79–1.06) | 0.230 |  |
| Low birthweight (<2500g) | 1.30 (0.77–2.18) | 0.310 |  |
| Neonatal ICU admission | 1.29 (0.75–2.17) | 0.350 |  |
| Caregiver internalising disorder | 0.85 (0.60–1.21) | 0.380 |  |
| Any internalising disorder | 0.82 (0.50–1.31) | 0.420 |  |
| Breastfeeding | 0.77 (0.41–1.49) | 0.430 |  |
| Other pregnancy complication | 1.29 (0.68–2.37) | 0.430 |  |
| Maternal alcohol use in pregnancy | 1.14 (0.78–1.66) | 0.490 |  |
| Executive function (z-score) | 1.03 (0.87–1.23) | 0.590 |  |
| Recent DSH/suicidal talk | 1.15 (0.58–2.20) | 0.670 |  |
| Gestational diabetes | 0.87 (0.42–1.69) | 0.690 |  |
| IQ (z-score) | 0.97 (0.80–1.17) | 0.720 |  |
| Race (white vs non-white) | 1.04 (0.74–1.48) | 0.810 |  |
| Childhood deprivation (z-score) | 0.99 (0.83–1.18) | 0.930 |  |
| Sex (female) | 1.01 (0.69–1.48) | 0.960 |  |
| Maternal smoking in pregnancy | 0.99 (0.65–1.49) | 0.970 |  |

***Sample.*** *Restricted to participants who attempted suicide (N=287): high-lethality attempters (lethality ≥2 on the C-SSRS, n=91) vs low-lethality attempters (score 0–1, n=196).*

***Analysis.*** *Single-predictor weighted logistic regression pooled across multiple imputation datasets, adjusted for sex and age (and ancestry principal components for genetic models). Cox regression was not included because all participants experienced the event (attempt); quasi-Poisson was not included because the outcome is binary.*

***Significance.*** ** p<0.05, ** p<0.01, *** p<0.001 (uncorrected).*

***Interpretation.*** *PRS-depression and PRS-anxiety distinguished medically serious from less severe attempts. Several perinatal indicators (UTI in pregnancy, eclampsia) were associated with higher lethality. Counterintuitively, caregiver suicide attempt and caregiver thought disorder were inversely associated with offspring lethality within the attempter subgroup, possibly reflecting earlier identification and protective intervention in families with known psychiatric history; caution is warranted given the limited sample size.*

***Caution.*** *Statistical power is limited (N=287). Non-significant estimates should not be interpreted as evidence of no association.*

**Table S20. Ideation-Restricted Sensitivity Analysis: Attempters vs Ideators-Without-Attempt**

| **Variable** | **Single-predictor OR (95% CI)** | **Multivariable OR (95% CI)** |
| --- | --- | --- |
| Sex (female) | 2.06 (1.66–2.55)***††† | 1.75 (1.23–2.56) |
| Caregiver suicide attempt | 1.68 (1.29–2.18)***††† | 1.84 (1.11–2.94) |
| Any externalising disorder | 1.50 (1.15–1.94)**†† | 1.84 (1.17–2.97) |
| Gestational diabetes | 4.00 (2.32–7.23)***††† | 4.78 (1.84–16.66) |
| Maternal alcohol use in pregnancy | 1.96 (1.53–2.50)***††† | 1.58 (1.02–2.46) |
| Breastfeeding | 1.50 (1.06–2.16)*† | 2.06 (1.07–4.09) |
| Neonatal ICU admission | 0.89 (0.64–1.22) | 0.56 (0.30–0.98) |
| Maternal smoking in pregnancy | 2.23 (1.70–2.92)***††† | 1.58 (0.97–2.71) |
| UTI in pregnancy | 1.83 (1.42–2.36)***††† | 1.26 (0.78–2.09) |
| PRS Anxiety (z-score) | 1.37 (1.22–1.55)***††† | 1.13 (0.81–1.68) |
| Caregiver thought disorder | 1.83 (1.36–2.47)***††† | 1.05 (0.57–1.92) |
| PRS Depression (z-score) | 1.33 (1.18–1.51)***††† | 1.00 (0.64–1.54) |
| Childhood threat (z-score) | 1.38 (1.25–1.52)***††† | 1.03 (0.85–1.25) |
| Childhood deprivation (z-score) | 1.21 (1.09–1.34)***††† | 1.03 (0.86–1.26) |
| Caregiver internalising disorder | 1.47 (1.19–1.81)***††† | 0.83 (0.54–1.27) |
| PRS Wellbeing (z-score) | 0.77 (0.69–0.87)***††† | 0.82 (0.65–1.00) |
| IQ (z-score) | 0.82 (0.74–0.91)***††† | 0.88 (0.72–1.08) |
| PRS Suicide Attempt (z-score) | 1.24 (1.08–1.41)**†† | 0.98 (0.63–1.47) |
| Race (white vs non-white) | 1.18 (0.96–1.44) | 1.34 (0.87–2.13) |
| Any internalising disorder | 1.29 (0.96–1.73) | 0.88 (0.55–1.41) |
| Recent DSH/suicidal talk | 1.15 (0.76–1.73) | 1.54 (0.67–3.56) |
| Low birthweight (<2500g) | 1.11 (0.81–1.52) | 0.99 (0.52–1.89) |
| Caregiver externalising disorder | 1.09 (0.26–4.14) | 0.23 (—) |
| Prenatal care ≥8 visits | 1.09 (0.87–1.37) | 1.02 (0.65–1.51) |
| PRS Suicide Death (z-score) | 1.07 (0.92–1.25) | 0.86 (0.64–1.18) |
| Executive function (z-score) | 0.99 (0.89–1.10) | 1.02 (0.81–1.32) |
| Other pregnancy complication | 0.98 (0.67–1.43) | 0.61 (0.28–1.26) |
| Eclampsia | 0.97 (0.74–1.26) | 0.92 (0.56–1.41) |
| Prematurity | 0.90 (0.67–1.21) | 1.03 (0.59–1.66) |

***Sample.*** *Restricted to participants with lifetime suicidal ideation (N=760): attempters (n=309) vs ideators without attempt (n=451). Ideation source: C-SSRS lifetime items 1–5.*

***Single-predictor.*** *Weighted logistic regression pooled across multiple imputation datasets, adjusted for sex and age (and ancestry principal components for genetic models). * p<0.05, ** p<0.01, *** p<0.001 (uncorrected); † FDR<0.05, †† FDR<0.01, ††† FDR<0.001 (Benjamini–Hochberg).*

***Multivariable.*** *Bootstrap with 1,000 iterations on the log-odds scale; 95% CI from the 2.5th–97.5th percentiles, exponentiated. All predictors entered simultaneously. Events per variable (EPV) = 10.7.*

***Robust predictors.*** *Predictors with multivariable bootstrap 95% CI excluding 1.0 (associated with attempts after mutual adjustment within the ideator subgroup): maternal alcohol use in pregnancy, breastfeeding, neonatal ICU admission, gestational diabetes, sex (female), caregiver suicide attempt, and any externalising disorder.*

***Interpretation.*** *Predictors significant in both the main analysis (Table 2) and the ideation-restricted analysis are interpreted as associated with the transition from ideation to action; those significant only in the main analysis are more likely shared with ideation risk.*

***Caution.*** *Ideation was ascertained concurrently with the outcome at Wave 3 (C-SSRS lifetime recall). Temporal ordering between ideation onset and attempt cannot be established; results should be interpreted as cross-sectional differentiation rather than prospective prediction of the ideation-to-action transition.*

**Supplementary Figures**

**Figure S1. Directed Acyclic Graph (DAG) of Hypothesised Causal Structure Across Temporal Layers** (Yellow indicates a new analytical figure added in revision.)


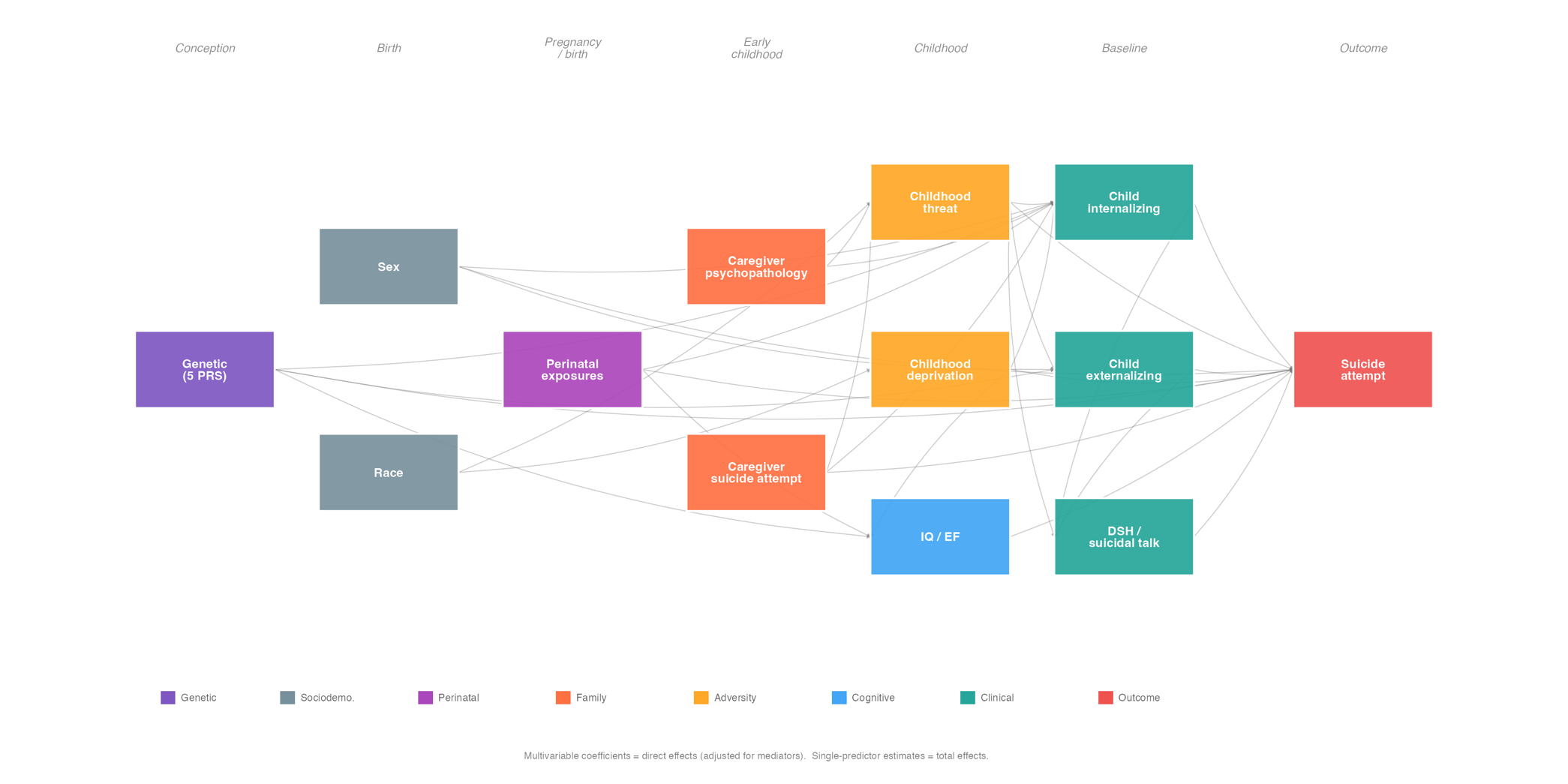


Conceptual model of relationships among risk factor domains, organised by temporal layer: genetic (conception) → perinatal → sociodemographic and family (early childhood) → adversity and cognition (childhood) → clinical (baseline) → outcome (suicide attempt at follow-up). Solid arrows depict hypothesised causal directions; double-headed arrows indicate bidirectional or correlational relationships. The DAG clarifies which variables function as confounders versus potential mediators in the multivariable models. Coefficients of upstream variables (e.g., adversity) represent direct effects, attenuated by adjustment for downstream mediators (e.g., psychopathology); single-predictor models capture total effects.

**Figure S2. Spearman Correlation Heatmap of Baseline Risk Factors**

*
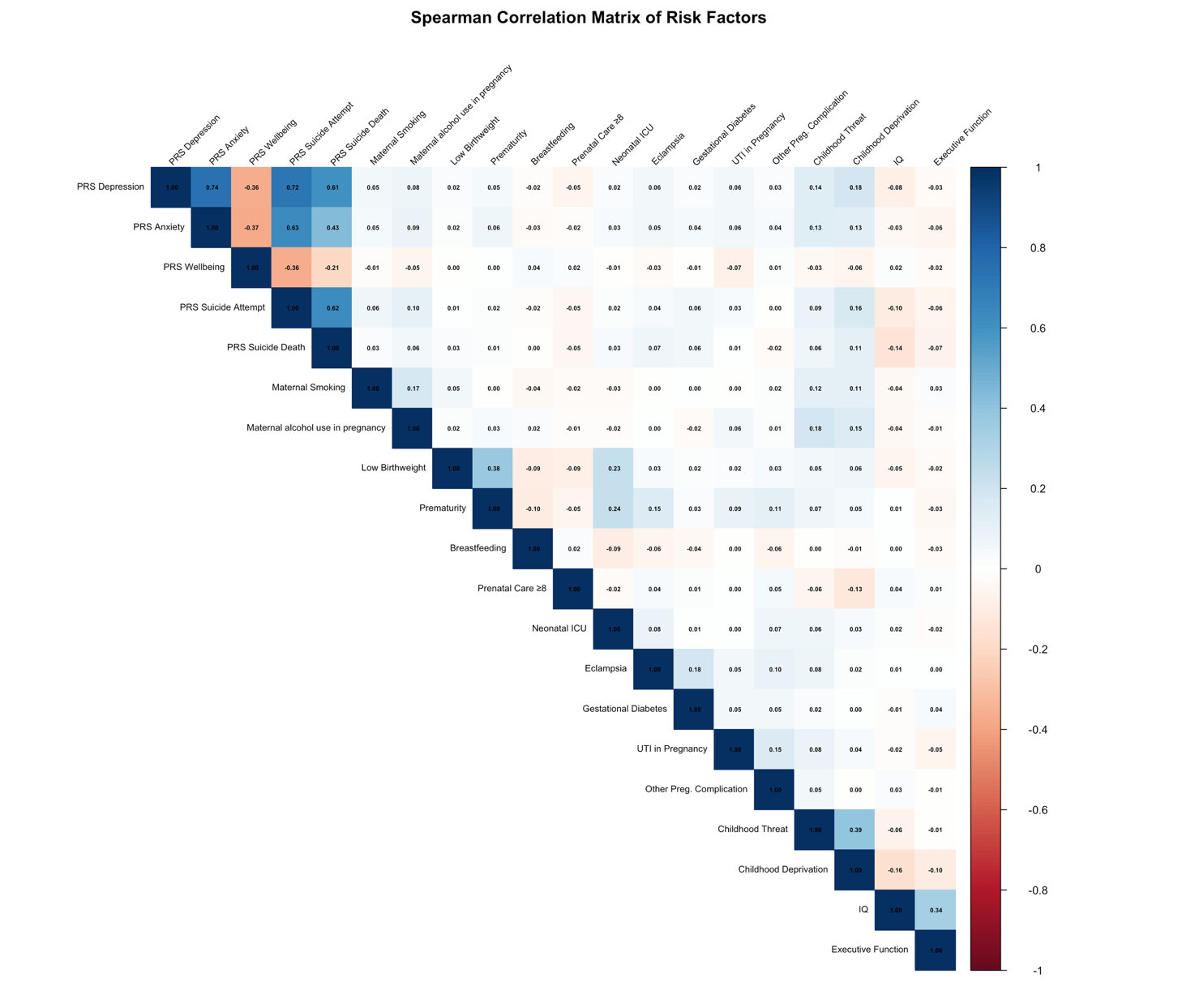
*

Spearman rank correlations among all baseline predictors entered in multivariable models (N=2,060). Colour intensity indicates the magnitude and direction of correlation; values are displayed within cells. Median correlation r=0·05; 93·7% of pairwise correlations had |r|<0·3. Higher correlations (|r|>0·5) were observed exclusively among polygenic risk scores (range 0·61–0·75), consistent with shared genetic architecture across psychiatric phenotypes. All GVIF values were below 5 (only PRSs >2; Table S2), indicating acceptable collinearity.

**Figure S3. Kaplan–Meier Cumulative Incidence of Suicide Attempts Stratified by Caregiver Suicide Attempt History**

*
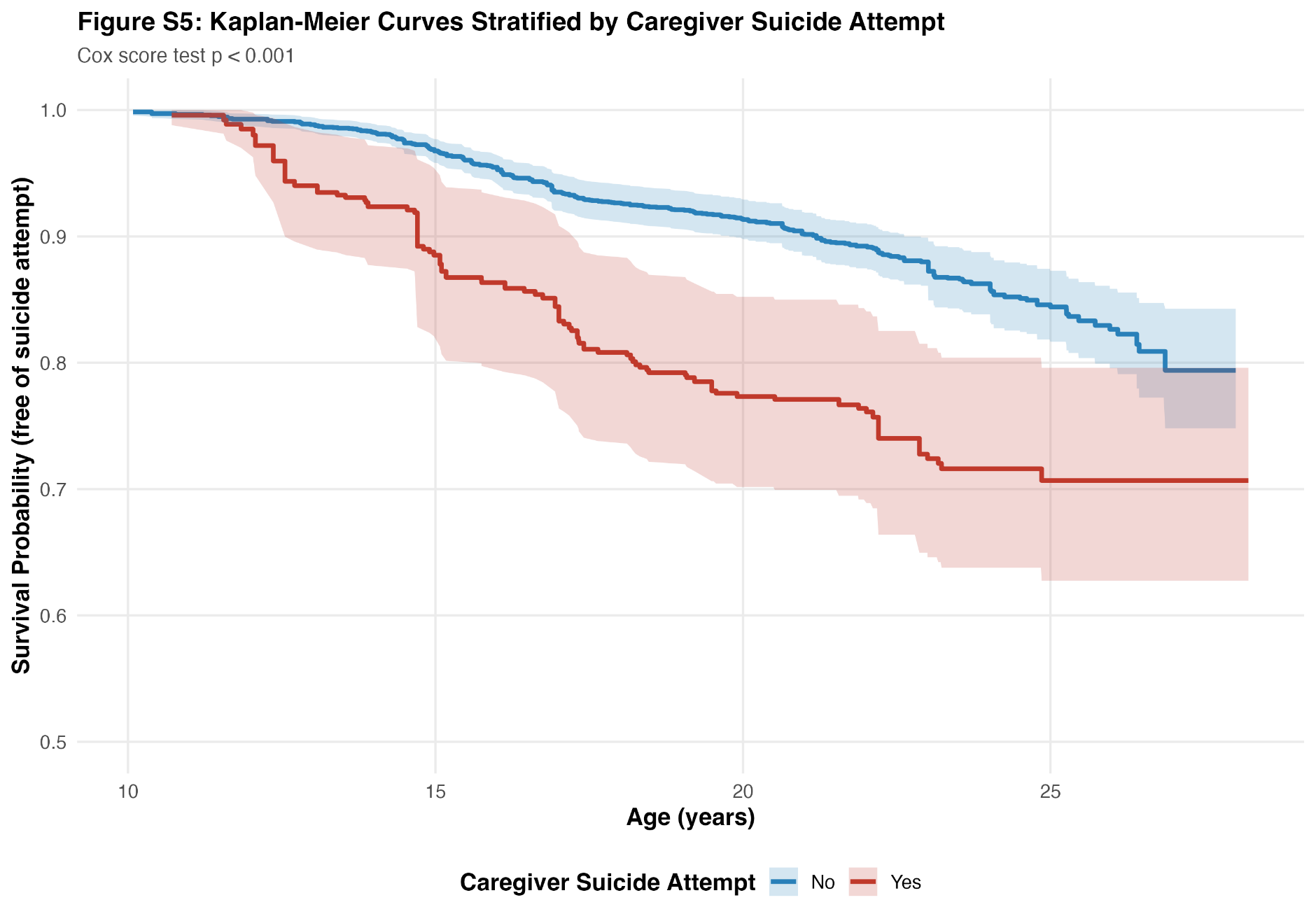
*

Cumulative incidence curves with age as the time scale, stratified by caregiver lifetime suicide attempt (yes vs no) at baseline. Shaded bands indicate 95% confidence intervals. Curves are weighted using CBPS weights. Log-rank test compared distributions between groups (p<0·001). Offspring of suicide attempters reached the 10% incidence threshold 6·5 years earlier than offspring of non-attempters (age 14·7 vs 21·2 years).

**Figure S4. Kaplan–Meier Cumulative Incidence of Suicide Attempts Stratified by Childhood Threat Exposure**

*
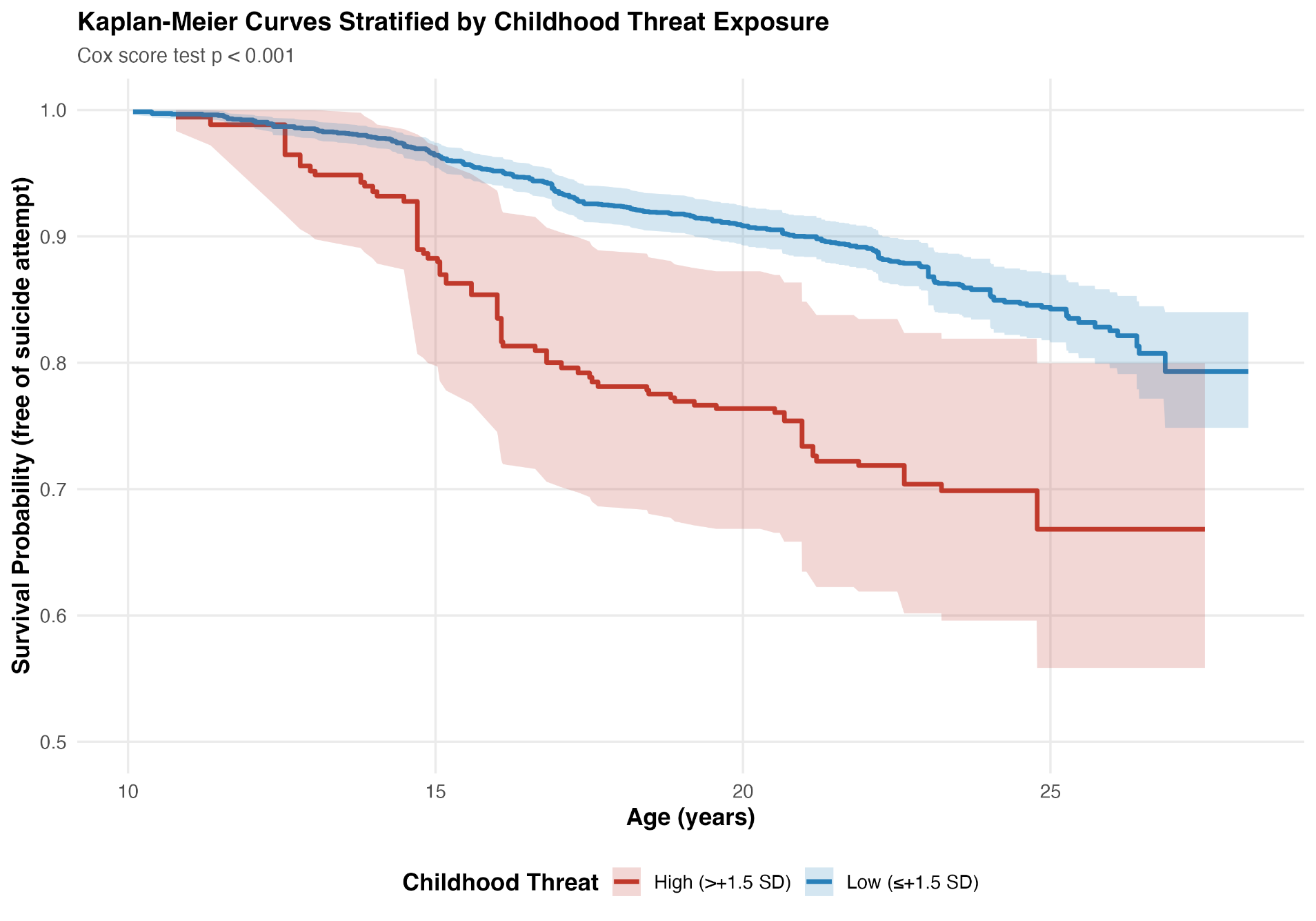
*

Cumulative incidence curves stratified by childhood threat exposure (high: >1 SD above mean vs low). Shaded bands indicate 95% confidence intervals. Curves are weighted using CBPS weights. Log-rank test compared distributions between groups (p<0·001). Participants with high threat exposure reached the 10% incidence threshold 6·1 years earlier (age 16·9 vs 23·0 years).

**Figure S5. Kaplan–Meier Cumulative Incidence of Suicide Attempts Stratified by Baseline Externalising Disorders**

*
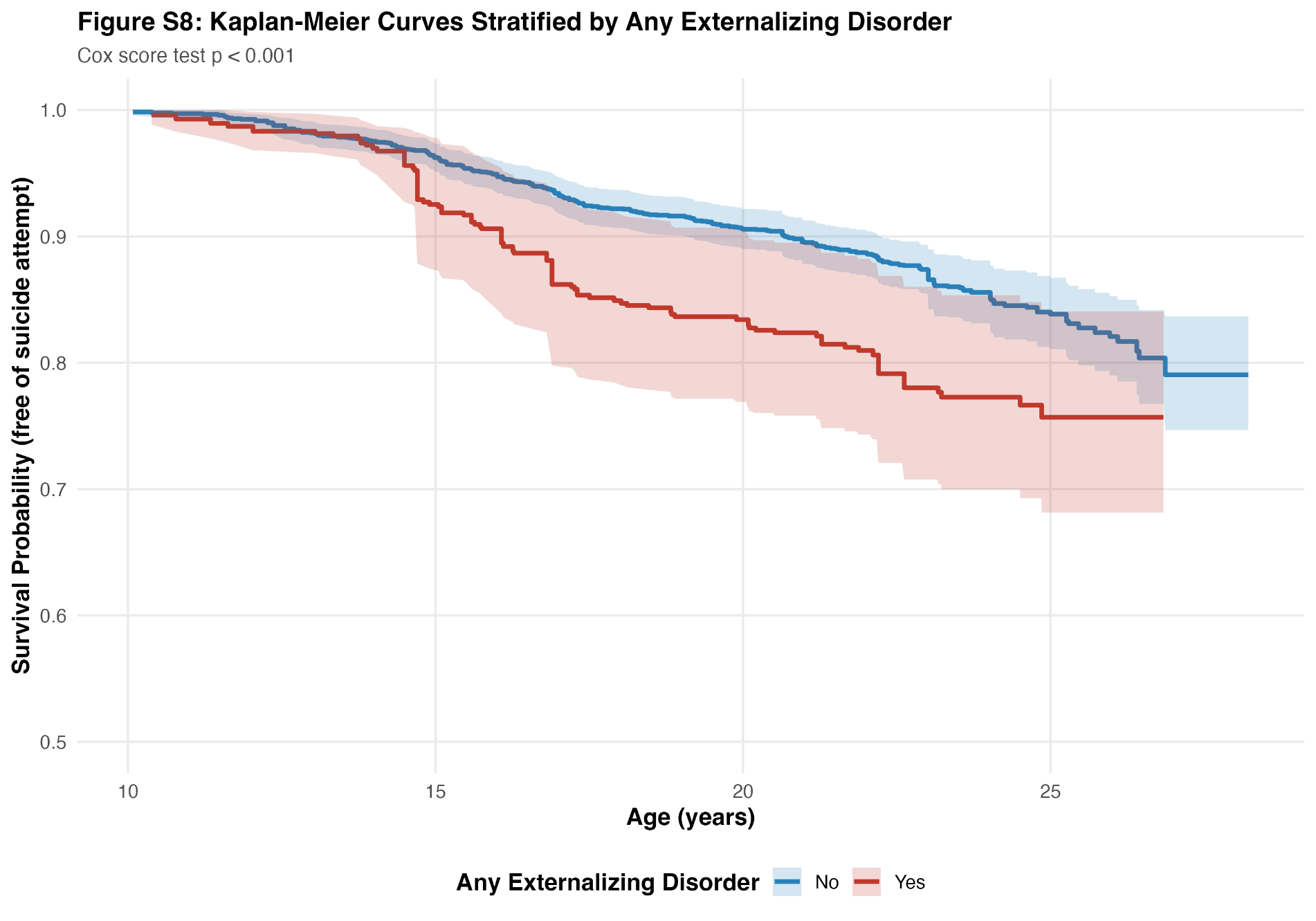
*

Cumulative incidence curves stratified by baseline externalising disorders (any vs none). Shaded bands indicate 95% confidence intervals. Curves are weighted using CBPS weights. Log-rank test compared distributions between groups (p<0·001). Participants with externalising disorders at baseline reached the 10% incidence threshold 4·6 years earlier (age 16·1 vs 20·7 years).

**Figure S6. Kaplan–Meier Cumulative Incidence of Suicide Attempts Stratified by Maternal Alcohol Use in Pregnancy**

*
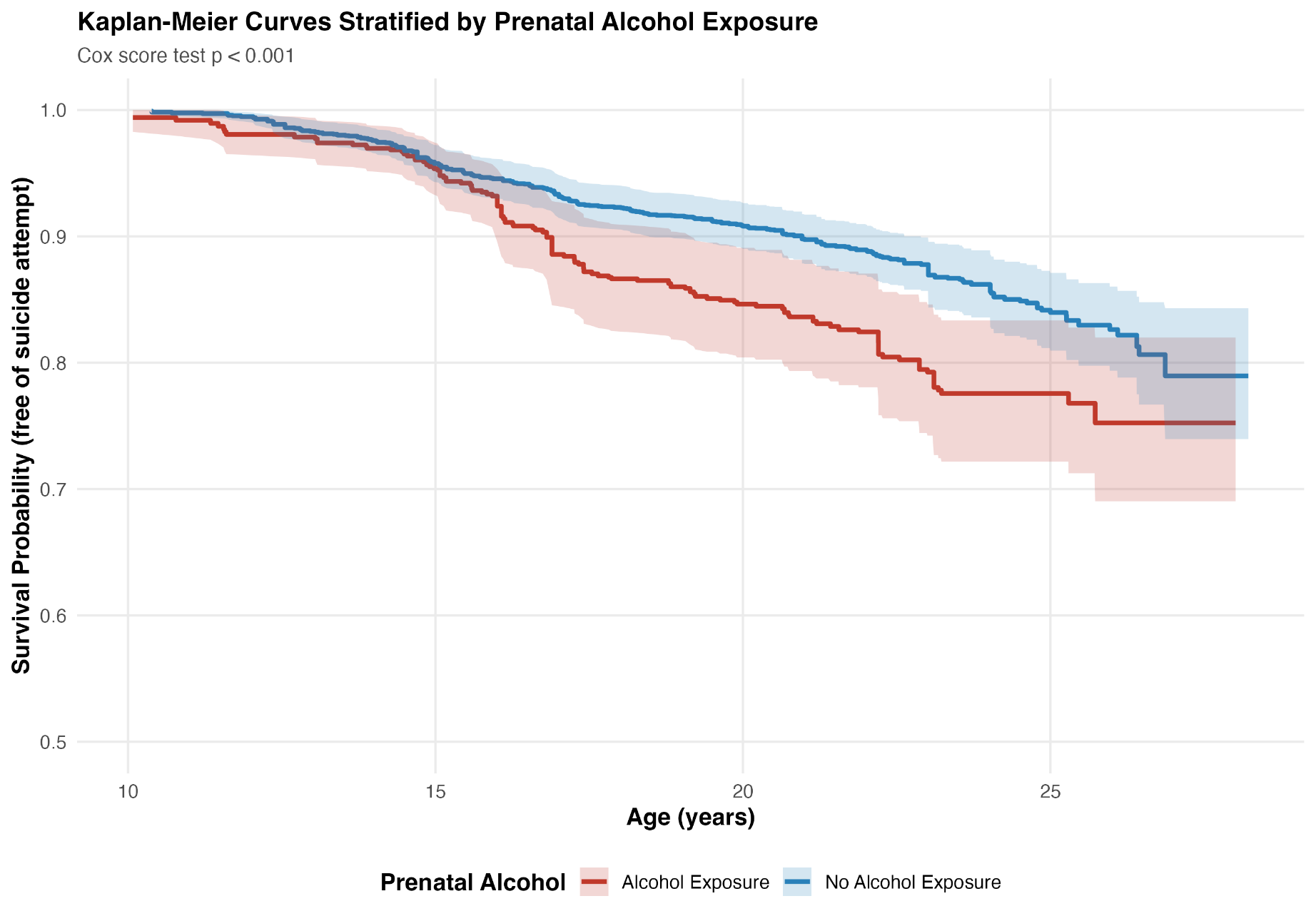
*

Cumulative incidence curves stratified by maternal alcohol use in pregnancy (any vs none). Shaded bands indicate 95% confidence intervals. Curves are weighted using CBPS weights. The figure illustrates the earlier-onset pattern observed in offspring of mothers who consumed alcohol during pregnancy, particularly relevant for high-lethality attempts (HR 1·92, 95% CI 1·12–3·26; Table S18).
